# Histopathology underlying environmental enteric dysfunction in a cohort study of undernourished children in Bangladesh, Pakistan, and Zambia compared with United States children^☆^

**DOI:** 10.1016/j.ajcnut.2024.02.028

**Published:** 2024-09-17

**Authors:** Paul Kelly, Kelley VanBuskirk, David Coomes, Samer Mouksassi, Gerald Smith, Zehra Jamil, Md Shabab Hossain, Sana Syed, Chelsea Marie, Phillip I Tarr, Peter B Sullivan, William A Petri, Donna M Denno, Tahmeed Ahmed, Mustafa Mahfuz, S Asad Ali, Sean R Moore, I Malick Ndao, Guillermo J Tearney, Ömer H Yilmaz, Shyam S Raghavan, Christopher A Moskaluk, Ta-Chiang Liu, EEDBI Consortium, Kumail Ahmed, Sheraz Ahmed, Md. Ashraful Alam, S.M. Khodeza Nahar Begum, Ellen Besa, Kanta Chandwe, Miyoba Chipunza, Subhasish Das, Lee A. Denson, Shah Mohammad Fahim, Md. Amran Gazi, Md. Mehedi Hasan, Aneeta Hotwani, Junaid Iqbal, Najeeha Talat Iqbal, Sadaf Jakhro, Furqan Kabir, Sarah Lawrence, Barbara J. Mann, Ramendra Nath Mazumder, Waheeda Memon, Brooks Morgan, Victor Mudenda, Chola Mulenga, Monica Mweetwa, Abdul Khalique Qureshi, Masudur Rahman, Najeeb Rahman, Kamran Sadiq, Shafiqul Alam Sarker, Fayaz Umrani, Kanekwa Zyambo

**Affiliations:** 20 Department of Paediatrics and Child Health, Aga Khan University, Karachi, Pakistan; 21 International Centre for Diarrhoeal Disease Research, Bangladesh, Dhaka, Bangladesh; 22 Department of Pathology, Bangladesh Specialized Hospital, Dhaka, Bangladesh; 23 Tropical Gastroenterology & Nutrition group, University of Zambia School of Medicine, Lusaka, Zambia; 24 Division of Pediatric Gastroenterology, Hepatology, and Nutrition, Cincinnati Children's Hospital Medical Center, Cincinnati, OH, USA; 25 Department of Biological and Biomedical Sciences, Aga Khan University, Karachi, Pakistan; 26 Department of Pediatrics, University of Washington School of Medicine, Seattle, WA, USA; 27 Department of Medicine, University of Virginia, Charlottesville, Virginia, USA; 28 Department of Epidemiology, University of Washington School of Public Health, Seattle, USA; 29 Department of Gastroenterology, Sheikh Russel National Gastroliver Institute and Hospital, Dhaka, Bangladesh; 1 Blizard Institute, Barts & The London School of Medicine, Queen Mary University of London, London, United Kingdom; 2 Department of Global Health, University of Washington School of Public Health, Seattle, WA, United States; 3 Department of Epidemiology, University of Washington School of Public Health, Seattle, WA, United States; 4 Certara, Princeton, NJ, United States; 5 Cytel, Vancouver, British Columbia, Canada; 6 Department of Biological and Biomedical Sciences, Aga Khan University, Karachi, Pakistan; 7 Nutrition Research Division, International Centre for Diarrhoeal Disease Research, Bangladesh, Dhaka, Bangladesh; 8 Department of Pediatrics, University of Virginia School of Medicine, Charlottesville, VA, United States; 9 Department of Medicine, University of Virginia School of Medicine, Charlottesville, VA, United States; 10 Department of Pediatrics, Washington University School of Medicine, St. Louis, MO, United States; 11 Department of Paediatrics, Children’s Hospital, University of Oxford, Oxford, United Kingdom; 12 Department of Pediatrics, University of Washington School of Medicine, Seattle, WA, United States; 13 Department of Paediatrics and Child Health, Aga Khan University, Karachi, Pakistan; 14 Department of Pathology, Harvard Medical School, Boston, MA, United States; 15 Department of Pathology, Massachusetts General Hospital, Boston, MA, United States; 16 Department of Pathology, University of Virginia School of Medicine, Charlottesville, VA, United States; 17 Department of Pathology and Immunology, Washington University School of Medicine, St. Louis, MO, United States

**Keywords:** enteropathy, stunting, Paneth cells, goblet cells, villi, crypts, intestine, global health, malnutrition, child health

## Abstract

**Background:**

Environmental enteric dysfunction (EED) is an asymptomatic intestinal disorder associated with growth impairment, delayed neurocognitive development, and impaired oral vaccine responses.

**Objectives:**

We set out to develop and validate a histopathologic scoring system on duodenal biopsies from a cohort study of children with growth failure in Bangladesh, Pakistan, and Zambia (“EED”) with reference to biopsies from United States children with no clinically reported histologic pathology (referred to hereafter as “normal”) or celiac disease.

**Methods:**

Five gastrointestinal pathologists evaluated 745 hematoxylin and eosin slide images from 291 children with EED (mean age: 1.6 y) and 66 United States children (mean age: 6.8 y). Histomorphologic features (i.e., villus/crypt architecture, goblet cells, epithelial and lamina propria acute/chronic inflammation, Brunner’s glands, Paneth cells, epithelial detachment, enterocyte injury, and foveolar metaplasia) were used to score each histopathologic slide. Generalized estimating equations were used to determine differences between EED, normal, and celiac disease, and receiver operating characteristic curves were used to assess predictive value.

**Results:**

Biopsies from the duodenal bulb showed higher intramucosal Brunner’s gland scores and lower intraepithelial lymphocyte scores than from the second or third parts of the duodenum (D2/3), so only D2/3 were included in the final analysis. Although 7 parameters differed significantly between EED and normal biopsies in regression models, only 5 (blunted villus architecture, increased intraepithelial lymphocytosis, goblet cell depletion, Paneth cell depletion, and reduced intramucosal Brunner’s glands) were required to create a total score percentage (TSP-5) that correctly identified EED against normal biopsies (AUC: 0.992; 95% CI: 0.983, 0.998). Geographic comparisons showed more severe goblet cell depletion in Bangladesh and more marked intraepithelial lymphocytosis in Pakistan.

**Conclusions:**

This scoring system involving 5 histologic parameters demonstrates very high discrimination between EED and normal biopsies, indicating that this scoring system can be applied with confidence to studies of intestinal biopsies in EED.

## Introduction

There is emerging consensus that environmental enteric dysfunction (EED) is a major contributor to childhood malnutrition [[1], [2], [3]], neurocognitive impairment [4], micronutrient deficiencies [5], and poor responses to oral vaccines [6] in low- and middle-income countries (LMICs). EED likely develops in children as a response to a heavy burden of asymptomatic polymicrobial intestinal infections [[7], [8], [9]] and possibly as an adaptation to them [10]. EED shares many features with enteropathies caused by infectious, autoimmune, and drug-induced processes, including villus blunting, inflammation, increased mucosal permeability, and epithelial damage [11]. EED impairs gut barrier function [12], digestion [13], and nutrient absorption [14], which have massive impacts on child health on a global scale, especially poor linear growth, which is a risk factor for chronic noncommunicable disease later in life. Short stature is also associated with economic disadvantage, with a 4% decrease in wages for every 1 cm reduced height in males and 6% in females [15]. There also exists intergenerational risk of stunting transmission from mother to child [4]. The scale of this problem—32% of sub-Saharan African and South Asian children were stunted in 2020 [16]—justifies strenuous efforts to understand the pathophysiology of EED [17] and the enteropathy that underlies it. In addition, it is now clear that stunting is highly refractory to nutritional interventions [18], underlining the need for new therapeutic approaches targeting underlying etiologies such as EED.

However, our understanding of EED in children is constrained by a lack of study of the target tissue—the small bowel—due to ethical challenges in obtaining small intestinal biopsies from children with this disorder [19]. In 2015, the Bill and Melinda Gates Foundation initiated the EED Biopsy Initiative (EEDBI) Consortium to study EED through intestinal biopsy protocols in Bangladesh, Pakistan, and Zambia [20]. A first step toward developing a scoring system for such biopsies has already been published [21]. We now report a histopathology score-based analysis of these EED biopsies compared with those obtained from United States children with celiac disease or with no mucosal histologic abnormality, hereafter referred to as “normal” biopsies. We set out to test the hypothesis that the pathology underlying EED varies across geographies and to address the following questions. Which histology features are peculiar to EED? Is EED a patchy disorder? Must biopsies for EED assessment be collected from a specific duodenal site? How much do technical histologic preparation parameters matter when comparing biopsy scores across populations? Can we develop a simple and easy-to-use scoring system that accurately discriminates between EED, normal, and celiac biopsies?

## Methods

The 5 studies that contributed to this analysis have been described in the first paper in this series [20], which explains inclusion and exclusion criteria. The studies across the 5 settings were carried out from March 1, 2016 to September 13, 2019 [20]. Briefly, pediatric biopsies were obtained in countries with a high prevalence of stunting and EED (Bangladesh, Pakistan, and Zambia). The children were from low-income, socially disadvantaged urban (Dhaka, Lusaka) or rural (Matiari) communities. The Biomarkers of Environmental Enteropathy in Children (BEECH) study recruited Zambian children with wasting (weight-for-length *z*-score < −2) or stunting (length-for-age *z*-score [LAZ] < −2). The Bangladesh Environmental Enteric Dysfunction (BEED) study included children with stunting and “at risk of stunting” (LAZ ≥ −2 but <−1). The Study of Environmental Enteropathy and Malnutrition (SEEM) study included Pakistani children with wasting. Duodenal biopsies representing EED were obtained at 3 study centers from undernourished children who were unresponsive to nutritional interventions (as described in [20] and [22]) and without other identifiable medical causes of growth faltering. Biopsies either lacking histological abnormality or from children with clinical, histologic, and serologic manifestations of celiac disease were obtained from Cincinnati Children’s Hospital Medical Center (CCHMC), Cincinnati, OH, United States, and the University of Virginia Children’s Hospital, Charlottesville, VA, United States. Biopsies at the United States centers were obtained from children presenting for endoscopy for clinical indications. They were considered not to have EED, and those either diagnosed with celiac disease or with normal biopsies were taken as non-EED comparison groups. In an attempt to obtain control (“normal”) tissues from children residing in EED endemic settings, biopsy sections that were clinically read as normal were obtained from the histopathology archives at Aga Khan University (AKU) Hospital Karachi, referred to hereafter as “AKU archival.”

### Ethical approvals

Ethical approval was obtained from AKU Ethics Review Committee (3836-Ped-ERC-15), Institutional Review Board (IRB) of International Centre for Diarrhoeal Disease Research, Bangladesh (PR-16007), University of Zambia Biomedical Research Ethics Committee (006-02-16), University of Virginia IRB (19466), and CCHMC IRB (2016-0387). Exemption was received from the University of Washington IRB (STUDY00013442) and the Washington University IRB (201801207). All children were biopsied only after fully informed, written consent was obtained, and the studies were conducted in full accordance with the Declaration of Helsinki and Good Clinical Practice.

### Scoring process

A total of 679 hematoxylin and eosin (H&E) slides from 291 children with EED, 1 slide from each of 66 children from the United States, and a further 7 children with “AKU archival” biopsies were evaluated. H&E slides were scanned and uploaded to a telepathology platform as previously described [21]. Five independent pathologists convened to standardize scoring criteria using a previously published scoring system [21] (Supplemental Table 1 and Supplemental Atlas). The scoring system consisted of 11 histologic parameters selected to cover a broad range of histopathologic processes without prior judgment about which features might be discriminating. Each parameter was scored on a scale of 0–3 or 0–4, or as nonscorable (missing) if histologic preparation precluded assessment. H&E slide images were randomly allocated to ≥2 pathologists (Supplemental Table 2). Scoring was conducted independently and blinded to the recruiting center where the biopsies were collected.

Initially each slide image was scored by 3 pathologists, but as throughput increased, this number was reduced to 2 (Supplemental Table 2). Thus, 120 biopsies were scored by 3 pathologists, and 632 biopsies were scored by 2 pathologists. To validate scoring by 2 pathologists, we randomly selected a subset of 50 slide images that had been scored by 3 pathologists. Using this subset, we simulated dual read scores for those triread slide images by randomly selecting and dropping 1 pathologist’s scores. Summary statistics (range, median, mean, and SDs) of triread scores for each histologic parameter for the subset of 50 slide images were qualitatively comparable to those from the simulated dual-reads (data not shown). In addition, in an interim analysis of concordance among pathologists, overall weighted percent agreement was 97.5% and Gwet’s agreement coefficient was 0.95 across all histologic parameters (Supplemental Table 6).

A final consensus score for each histologic parameter was calculated as a mean of the pathologists’ scores for that slide image. To assess technical issues (Supplemental Table 3), biopsies were also scored for orientation, staining quality, and crush/drying artifacts.

### Data analysis

Analyses were performed to investigate the following: *1*) whether histology differed between proximal (D1) and more distal (D2/D3) duodenal biopsies, *2*) whether histology differed between the 3 groups analyzed (EED, normal, and celiac participants), *3*) the extent to which technical limitations affected the differences observed, and *4*) whether histology differed between participants at the EED study centers, taking into account technical issues. Models for these aims were set up with the individual histologic parameter and the global index score (see below) as outcome and disease status, EED study center, or biopsy site as predictor. Multivariable models were adjusted for histology quality measurements—tissue orientation, staining quality, drying, and crush artifact were scored using the scale presented in Supplemental Table 3. Age was collinear with disease (i.e., there was no overlap in age between children in the EED and those in the United States centers). Age varied little across the EED cohort, and in models restricted to the EED cohorts, adjusting for it did not meaningfully change point estimates. Hence, age was not included in final multivariable models. In order to account for the fact that some patients had multiple slide images, generalized estimating equation (GEE) models were used. As a sensitivity analysis, linear mixed effect models were fit and results were compared with the results for the GEE models. Descriptive statistics of patient characteristics and histology summary statistics are presented as median and IQR. Statistical analyses were performed using R version 4.1.0 (R Foundation for Statistical Computing).

#### Index scores

Several index scores were developed to combine the different histologic parameters into one measure of histopathology in attempts to refine the previously published total score percent (TSP) using all 11 individual histology parameters [21]. Total score was a simple sum of scores for the 11 individual histologic parameters, as described in the original article on the histopathology scoring system [21]. However, the denominator needed to be adjusted for nonscorable values. To ensure better comparability of total score across slide images with variable numbers of nonscorable histology parameters, an alternative index score, TSP was calculated: the sum of individual parameter scores was divided by the total possible score for all scored parameters and multiplied by 100 to generate a percentage.

We additionally calculated the TSP-5, which was derived from the original TSP variable by including only the scores for the parameters found to be most discriminating for disease status and still producing a high AUC. We tested the TSP-5 against the original TSP by comparing their receiver operating characteristic (ROC) curves and confusion matrices for discriminating between disease states (EED and normal).

Separately, a weighted composite score was generated using histologic parameter weightings derived from logistic regression models in which EED or normal was the dependent variable and histologic parameters were independent variables. Imputation was used to handle responses of nonscorable for histologic parameters in computing these composite scores. To assess performance of these index scores in discriminating between EED and normal biopsies, the dataset was divided into training (80%) and testing (20%) sets, and 10 iterations were performed to derive ROC curves and confusion matrices for each index score using the “rsample” R package.

Information regarding previously published single-center data relevant to this multicenter histology analysis is provided elsewhere in this supplement issue [20].

## Results

**FIGURE 1:**
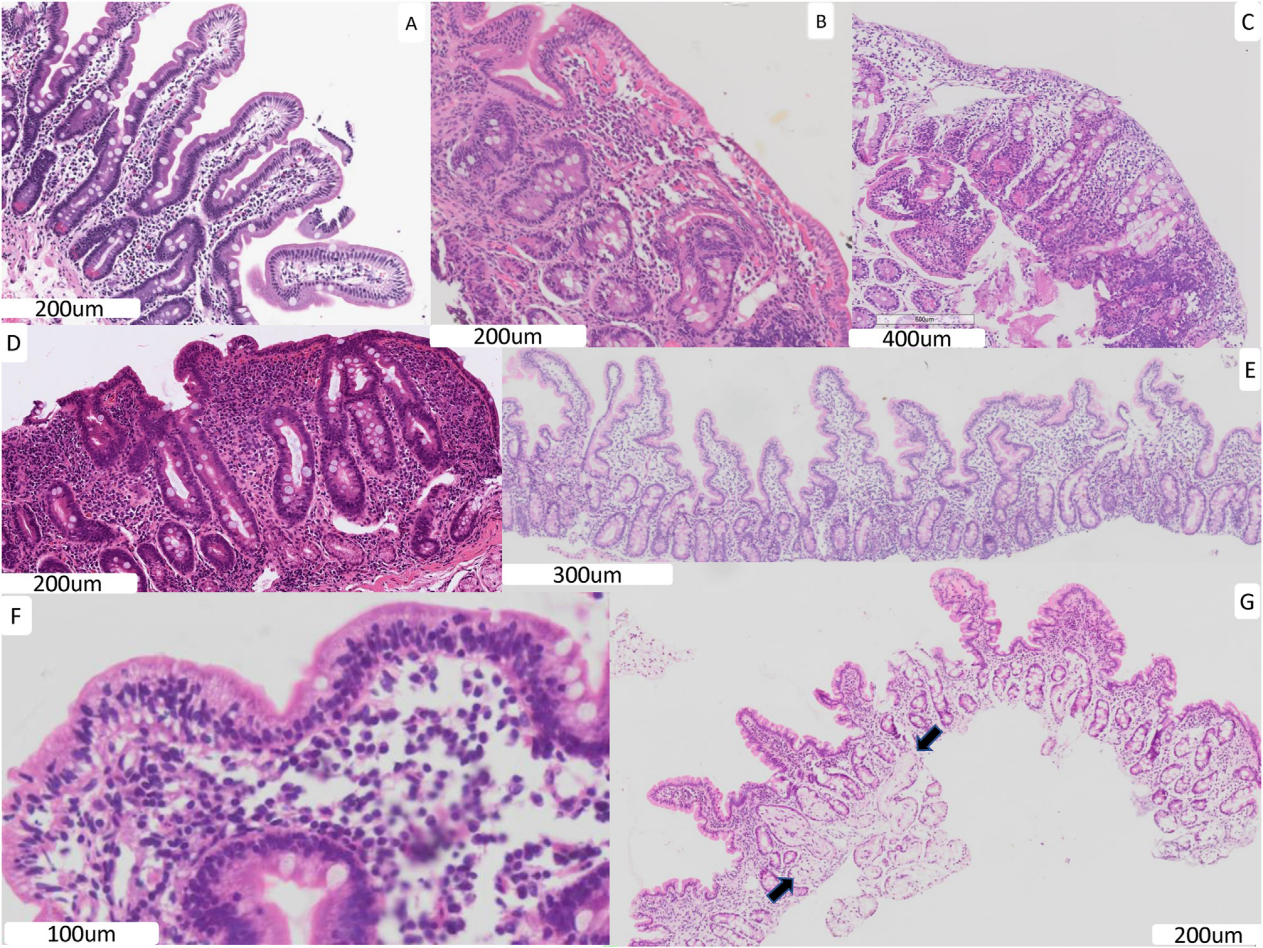
Representative images of biopsies from EED, normal biopsies, and celiac disease. (A) Biopsy from a healthy United States child. There are long finger-like villi with a villus height to crypt length ratio of ≥4. The epithelial cells have a tall columnar morphology, and goblet cells extend to the luminal surface. Highly eosinophilic Paneth cells are apparent at the base of the crypts. (B) Biopsy from a United States child with celiac disease. There is total loss of villus architecture (complete blunting) and the surface epithelial cells are markedly thinned. There is an increased mononuclear inflammatory cell infiltrate in the lamina propria. (C, D) Environmental enteric dysfunction (EED) biopsies with high scores across several domains. There is almost total loss of villus architecture. The surface epithelial cells are mostly cuboidal in morphology, and there is loss of goblet cells on the mucosal surface. These biopsies also show a loss of Paneth cells at the crypt bases and an increased mononuclear inflammatory cell infiltrate in the lamina propria. (E) Low magnification images of EED biopsy showing relatively well-preserved villus architecture. (F) EED biopsy with very high intraepithelial lymphocyte score. (G) Low magnification image of EED biopsy with intramucosal Brunner’s gland penetration (arrows).

A total of 745 H&E slide images from 357 children were evaluated (Table 1), together with 7 AKU archival slides. Children from the EED centers were generally in the second year of life, whereas children with either no pathologic abnormality detected (“normal”) or celiac disease were several years older. Biopsies from children with EED and celiac disease demonstrated villus blunting and other pathologic features compared with those from normal children (Figure 1). The AKU archival biopsies were also evaluated as a potential “local” comparison group. Though considered to have normal mucosal architecture on diagnostic pathology reports, 1 showed *Giardia intestinalis* trophozoites, and *Helicobacter pylori* was observed in 3. The TSP-5 (see below) measured in AKU archival biopsies (median: 41.5) was much closer to EED biopsies (median: 52.8) than normal biopsies from North America (median: 11.9). AKU archival biopsies were therefore not used in the remaining analyses.

**TABLE 1:** Clinical and demographic characteristics of children whose biopsies were included in this study.

Geography	SEEM	BEED	BEECH	CCHMC	UVa	CCHMC and UVa
	Pakistan	Bangladesh	Zambia	United States	United States	United States
Disease status	EED			“Normal”: no pathologic abnormality reported by diagnostic pathologist		Celiac disease
Biopsy strategy	D1 and D2/3, separate slides	D1 and D2/3, separate slides	D2/3 only	Mixed D1 and D2/3 slides	D2/3 only	Mixed D1 and D2/3 slides
No. of children	63	120	108	28	16	22
No. of biopsies	54 D1 180 D2/D3	120 D1 120 D2/D3	205 D2/D3	1 D1 22 D1/D2/D3 5 D2/D3	16 D2/D3	19 D1/D2/D3 3 D2/D3
Sex (% female)	30.2%	58.3%	50.9%	42.9%	50%	54.5%
Age^1^ (y), median (IQR)	1.7 (1.3, 1.8)	1.6 (1.4, 1.7)	1.6 (1.3, 1.8)	5.5 (4.2, 6.7)	12.1 (7.6, 15.6)	7.1 (5.2, 10.0)
Inclusion criteria	Children with wasting refractory to nutritional intervention	Children with stunting or “at risk of stunting” (LAZ < −1 but ≥ −2) refractory to nutritional intervention	Children with LAZ or WLZ consistently <−2 despite nutritional intervention	Children 1–18 y undergoing endoscopy for clinical indications	Children under 12 y undergoing endoscopy for clinical indications	Children under 18 y undergoing endoscopy to confirm celiac disease
HAZ/LAZ^2^, median (IQR)	−2.1 (−2.8, −1.5)	−3.2 (−3.7, −2.4)	−3.3 (−3.9, −2.8)	0.0 (−1.0, 1.2)	0.7 (−0.5, 1.6)	0.1 (−0.7, 0.4)
WAZ^2^, median (IQR)	−1.7 (−2.3, −1.2)	−3.1 (−3.6, −2.6)	−2.2 (−2.7, −1.8)	0.0 (0.0, 0.5)	0.3 (0.2, 0.6)	0.5 (0.5, 0.9)
WHZ/WLZ^2^^,^^3^, median (IQR)	−1.0 (−1.4, −0.4)	−2.2 (−2.8, −1.8)	−0.8 (−1.3, −0.2)	−0.6 (−1.0, 0.0)	—	−0.6 (−0.6, 0.2)
BMI (kg/m^2^)^2^^,^^4^, median (IQR)	—	—	15.0 (14.7, 15.5)	15.4 (14.2, 16.5)	18.4 (16.2, 23.4)	15.4 (14.8, 16.5)

Abbreviations: BEECH, Biomarkers of Environmental and Enteropathy in Children; BEED, Bangladesh Environmental Enteric Dysfunction; BMI, body mass index; CCHMC, Cincinnati Children’s Hospital Medical Center; D1, first part of duodenum; D2/3 second/third parts of duodenum; EED, environmental enteric dysfunction; HAZ, height-for-age *z*-score; LAZ, length-for-age *z*-score; SEEM, Study of Environmental Enteropathy and Malnutrition; UVa, University of Virginia; WAZ, weight-for-age *z*-score; WHZ, weight-for-height *z*-score; WLZ, weight-for-length *z*-score.

1 At the time of biopsy

2 Measurements closest to the time of biopsy

3 WHZ/WLZ can only be calculated using WHO growth standards for children <5 y. All EED cohorts included children <5 y. CCHMC Celiac cohort included 4, UVa Celiac included 1, CCHMC Nondiagnostic included 10, and UVa Nondiagnostic included 0.

4 BMI is used as a measure of thinness (or overweight status) among children ≥2 y. All United States cohorts included children ≥2 y. BEECH cohort included 11 children ≥2 y, but BEED and SEEM did not.

**FIGURE 2:**
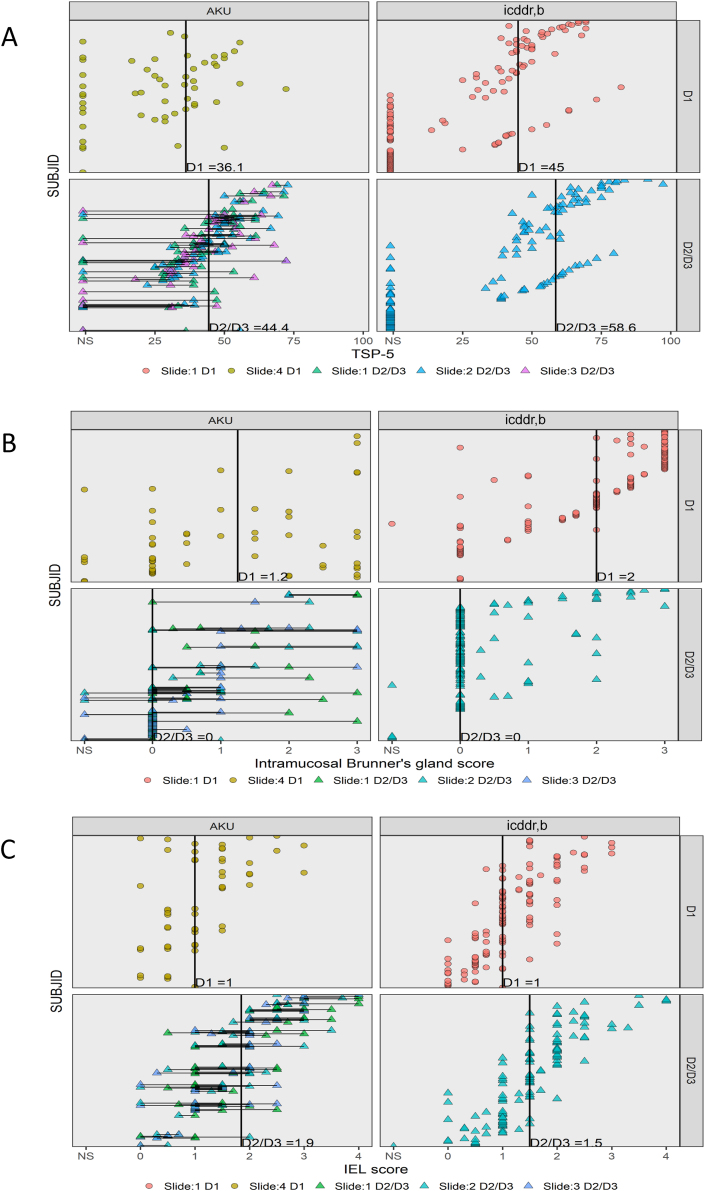
Biopsies from the first part of the duodenum differ in key respects from those from the second or third part. Scores of individual slides taken from biopsies in the duodenal bulb (D1) or the second or third part of the duodenum (D2/3) are displayed. Each point represents a score from an individual biopsy; biopsies taken from the same child are joined by a horizontal line. In each panel biopsies from AKU and icddr,b are shown in separate columns. The TSP-5 shows lower median TSP-5 scores in biopsies from both AKU and icddr,b compared with D2/3 biopsies (panel A). This is attributable to higher median intramucosal Brunner's gland scores (panel B) in D1 biopsies (which were then negatively scored for the calculation of TSP-5), and lower median intraepithelial lymphocyte scores in D1 biopsies (panel C). AKU, Aga Khan University; D1, first part of duodenum; D2/3 second/third parts of duodenum; EED, environmental enteric dysfunction; icddr,b, International Centre for Diarrhoeal Disease Research, Bangladesh; IEL, intraepithelial lymphocyte; SUBJID, participant ID; TSP-5, total score percent for 5 parameters.

EED biopsies taken from the first part of the duodenum demonstrated denser intramucosal Brunner’s gland penetration and fewer intraepithelial lymphocytes (IELs) compared with those from the second/third part (Figure 2). The difference in biopsy scores comparing biopsies taken from D1 to those from D2/3 is shown in Supplemental Table 4. To reduce variability and avoid bias, and because biopsies from Lusaka did not include D1, subsequent analysis included only biopsies from D2/3. This focus on D2/3 did not reduce the number of children contributing to the analysis.

### Histology parameters: EED compared with normal and celiac

The final scoring system is shown in Table 2. Summary statistics of all 11 histology parameters are presented by group in Table 3, along with the TSP-5, which is described below and in Table 2. Three histology parameters (acute inflammation, eosinophilic infiltration, foveolar metaplasia) had median scores of 0 across all groups (EED, celiac, and normal) and did not differ between groups (Table 3); these parameters were not considered further. Slide staining and biopsy orientation technical issues resulted in a moderate proportion of images not being able to be scored for Paneth cells and villus architecture that differed by study center (Table 3). Among those that could be scored, Paneth cell depletion was the most consistently abnormal feature identified in the EED slide images followed by chronic inflammation, villus architecture, IELs, and finally, goblet cell depletion, all of which except for chronic inflammation had low scores among the United States normal group. Although epithelial detachment had a mildly raised score among the EED cohort, it was similar to that of the United States normal cohort. Intramucosal Brunner’s glands were not prominent in the EED cohort but scored high among the United States cohorts. United States celiac slide images scored especially high for the IEL, villus architecture, and chronic inflammation parameters.

**TABLE 2:** Final histology scoring system showing the 5 parameters included in the TSP-5 and the 3 that were not included.

Included in TSP-5	Villus architecture	0: Majority of villi are >3 crypt lengths long	1: Villi are < 3 but >1 crypt lengths long, with abnormality involving ≤50% of mucosa	2: Villi are ≤3 but >1 crypt lengths long, with abnormality involving >50% of mucosa	3: Villi absent, or <1 crypt length long, with abnormality involving ≤50% of mucosa	4: Villi absent, or <1 crypt length long, with abnormality involving >50% of mucosa	NS: Not scorable
	Intraepithelial lymphocytes	0: No areas observed with epithelial/lymphocyte ratio ≥20%	1: Lymphocyte/ epithelial ratio >20%, but <50%, in <50% of mucosa	2: Lymphocyte/ epithelial ratio >20%, but <50% in >50% of mucosa	3: Lymphocyte/ epithelial ratio ≥50% in <50% of mucosa	4: Lymphocyte/ epithelial ratio ≥50% in >50% of mucosa	NS: Not scorable
	Goblet cell density depletion	0: Normal goblet cell density (≥1 goblet cell per 20 enterocytes) in all evaluable mucosal epithelial layer	1: Decreased goblet cells (<1/20 enterocytes) in 1%–25% of evaluable mucosal epithelium	2: Decreased goblet cells (<1/20 enterocytes) in 26%–50% of evaluable mucosal epithelium	3: Decreased goblet cells (<1/20 enterocytes) in 51%–75% of evaluable mucosal epithelium	4: Decreased goblet cells (<1/20 enterocytes) in 76%–100% of evaluable mucosal epithelium	NS: Not scorable
	Paneth cell density depletion	0: ≥5 Paneth cells/ crypt base, on average	1: 2–4 Paneth cells/crypt base, on average	2: <2 Paneth cell/crypt base, involving <50% of crypt bases	3: <2 Paneth cell/crypt, involving >50% of crypt bases	—	NS: Not scorable
	Intramucosal Brunner’s glands	3: None observed	2: 1 or 2 foci of intramucosal Brunner’s glands, none involving >5 crypt bases	1: 3–5 foci of intramucosal Brunner’s glands, none involving >5 crypt bases	0: >5 foci, or any area of intramucosal Brunner’s glands involving >5 crypt bases	—	NS: Not scorable
Not included in TSP-5	Chronic inflammation	0: No qualitative increase in MICs in lamina propria. Majority of villus bases contain <3 MICs across, on average	1: Increased MICs, based on villus base displaying 3–5 MICs across, on average	2: Increased MICs, based on villus base displaying 6–10 MICs across, on average	3: Increased MICs, based on villus base displaying >10 lymphocytes on average	—	NS: Not scorable
	Enterocyte injury	0: Majority of enterocytes (90%) show tall columnar morphology	1: Enterocytes show low columnar (<2:1 L:W ratio), cuboidal or flat morphology, in ≤50% of mucosa	2: Enterocytes show low columnar (<2:1 L:W ratio), cuboidal or flat morphology, in >50% of mucosa	3: Any area of mucosal erosion/ulceration	—	NS: Not scorable factor
	Epithelial detachment	0: Complete coverage of mucosal surface by epithelial cells	1: Surface epithelium missing or detached from <25% of mucosa	2: Surface epithelium missing or detached from 25%–50% of mucosa	3: Surface epithelium missing or detached from 51%–75% of mucosa	4: Surface epithelium missing or detached from >75% of mucosa	NS: Not scorable

This scoring system was developed and published in [18]. The scoring scheme for these histology parameters remains unchanged other than the reverse coding for intramucosal Brunner’s glands. This reverse coding update now reflects that low scores were associated with EED and high scores associated with normal (opposite of other histology parameters).

Abbreviations: MIC, mononuclear inflammatory cell; NS, not scorable: variable cannot be determined because of slide quality or other factor; TSP-5, total score percent of 5 parameters.

**TABLE 3:** Histopathology scores of participants by site and disease status.

	BEED *n* = 120 (120)	SEEM *n* = 63 (180)	BEECH *n* = 108 (205)	EED sites combined *n* = 291 (505)	UVa Celiac *n* = 2 (2)	CCHMC Celiac *n* = 20 (20)	United States Celiac combined *n* = 22 (22)	UVa Normal *n* = 16 (16)	CCHMC Normal *n* = 28 (28)	United States Normal combined *n* = 44 (44)
TSP-5 (0%–100%) % Not scorable	58.6 (47.2, 66.7) 33.3%	44.5 (36.7, 52.4) 0%	54.4 (47.1, 59.8) 3.7%	52.8 (44.4, 60.7) 15.1%	55.5 (52.8, 58.3) 0%	40.3 (30.6, 48.7) 0%	44.5 (31.3, 50.0) 0%	13.9 (7.6, 21.2) 0%	11.9 (7.2, 17.0) 0%	11.9 (7.2, 18.3) 0%
Goblet cell depletion (0–4) % Not scorable	2.0 (1.4, 2.5) 0%	1.0 (0.8, 1.3) 0%	1.5 (1.0, 2.0) 1.9%	1.5 (1.0, 2.0) 0.7%	0.4 (0.3, 0.4) 0%	0.5 (0.5, 1.0) 0%	0.5 (0.5, 1.0) 0%	0.3 (0.0, 0.6) 0%	0.0 (0.0, 0.3) 0%	0.0 (0.0, 0.5) 0%
Intramucosal Brunner’s glands (0–3) % Not scorable	0.0 (0.0, 0.5) 3.3%	0.2 (0.0, 1.0) 0%	0.0 (0.0, 0.2) 0.9%	0.0 (0.0, 0.5) 1.7%	1.0 (0.5, 1.5) 0%	3.0 (3.0, 3.0) 0%	3.0 (3.0, 3.0) 0%	3.0 (1.2, 3.0) 0%	2.4 (0.7, 3.0) 0%	3.0 (1.0, 3.0) 0%
Intraepithelial lymphocytes (0–4) % Not scorable	1.5 (1.0, 2.0) 0.8%	1.7 (1.2, 2.5) 0%	1.0 (0.5, 1.5) 0.9%	1.5 (1.0, 2.0) 0.7%	2.5 (2.2, 2.8) 0%	3.0 (1.9, 3.1) 0%	3.0 (2.0, 3.0) 0%	0.3 (0.0, 0.5) 0%	0.5 (0.0, 0.7) 0%	0.3 (0.0, 0.5) 0%
Paneth cell depletion (0–3) % Not scorable	3.0 (1.9, 3.0) 46.7%	0.7 (0.5, 1.0) 1.6%	2.0 (1.0, 3.0) 11.1%	1.5 (1.0, 3.0) 23.7%	1.1 (0.9, 1.3) 0%	0.5 (0.4, 0.8) 0%	0.5 (0.5, 0.9) 0%	0.3 (0.2, 0.6) 0%	0.0 (0.0, 0.5) 0%	0.1 (0.0, 0.5) 0%
Villus architecture (0–4) % Not scorable	2.0 (1.0, 3.0) 39.2%	2.1 (1.3, 3.0) 9.5%	2.5 (1.8, 3.3) 6.5%	2.0 (1.5, 3.1) 20.6%	4.0 (4.0, 4.0) 0%	3.2 (2.4, 4.0) 0%	3.8 (2.5, 4.0) 0%	0.5 (0.3, 0.7) 18.8%	0.3 (0.0, 0.5) 3.6%	0.5 (0.0, 0.5) 9.1%
Chronic inflammation (0–3) % Not scorable	1.5 (1.0, 1.5) 1.7%	1.3 (1.0, 1.6) 0%	1.5 (1.2, 2.0) 1.9%	1.5 (1.0, 1.8) 1.4%	2.2 (2.1, 2.4) 0%	2.0 (1.5, 2.0) 0%	2.0 (1.6, 2.0) 0%	1.0 (0.6, 1.0) 0%	0.8 (0.4, 1.0) 0%	1.0 (0.5, 1.0) 0%
Enterocyte injury (0–3) % Not scorable	0.3 (0.0, 0.5) 0%	0.3 (0.2, 0.5) 0%	0.2 (0.0, 0.5) 0.9%	0.3 (0.0, 0.5) 0.3%	1.1 (1.1, 1.2) 0%	0.8 (0.4, 1.1) 0%	1.0 (0.5, 1.2) 0%	0.3 (0.0, 0.3) 0%	0.0 (0.0, 0.1) 0%	0.0 (0.0, 0.3) 0%
Epithelial detachment (0–4) % Not scorable	1.0 (0.5, 1.0) 0%	1.0 (0.8, 1.2) 0%	1.0 (0.7, 1.2) 0%	1.0 (0.7, 1.2) 0%	0.8 (0.6, 0.9) 0%	1.0 (0.9, 1.1) 0%	1.0 (0.8, 1.0) 0%	1.0 (1.0, 1.6) 0%	0.7 (0.5, 1.0) 0%	0.8 (0.6, 1.0) 0%
Acute inflammation (0–3) % Not scorable	0.0 (0.0, 0.0) 0%	0.0 (0.0, 0.0) 0%	0.0 (0.0, 0.0) 0.9%	0.0 (0.0, 0.0) 0.3%	0.0 (0.0, 0.0) 0%	0.0 (0.0, 0.0) 0%	0.0 (0.0, 0.0) 0%	0.0 (0.0, 0.0) 0%	0.0 (0.0, 0.0) 0%	0.0 (0.0, 0.0) 0%
Foveolar cell metaplasia (0–3) % Not scorable	0.0 (0.0, 0.0) 0.8%	0.0 (0.0, 0.0) 0%	0.0 (0.0, 0.0) 0.9%	0.0 (0.0, 0.0) 0.7%	0.3 (0.2, 0.5) 0%	0.0 (0.0, 0.1) 0%	0.0 (0.0, 0.4) 0%	0.0 (0.0, 0.1) 0%	0.0 (0.0, 0.0) 0%	0.0 (0.0, 0.0) 0%
Eosinophil infiltration (0–3) % Not scorable	0.0 (0.0, 0.0) 0%	0.0 (0.0, 0.2) 0%	0.0 (0.0, 0.0) 0.9%	0.0 (0.0, 0.0) 0.3%	0.0 (0.0, 0.0) 0%	0.5 (0.2, 1.0) 0%	0.5 (0.0, 0.9) 0%	0.0 (0.0, 0.0) 0%	0.0 (0.0, 0.5) 0%	0.0 (0.0, 0.0) 0%

Data are shown as median (IQR). Scores exclude samples taken from the first part of duodenum. *n* refers to number of children (number of biopsies). For each histology parameter and the summative TSP-5, the possible range of scores is provided in the first column. The % not scorable represents the proportion of slide images that were determined to be not scorable due to technical slide quality issues by the scoring pathologists.

Abbreviations: BEECH, Biomarkers of Environmental and Enteropathy in Children; BEED, Bangladesh Environmental Enteric Dysfunction; CCHMC, Cincinnati Children’s Hospital Medical Center; EED, environmental enteric dysfunction; SEEM, Study of Environmental Enteropathy and Malnutrition; TSP-5, total score percent-5; UVa, University of Virginia.

### Development of a refined index score to differentiate between EED and normal biopsies

**FIGURE 3:**
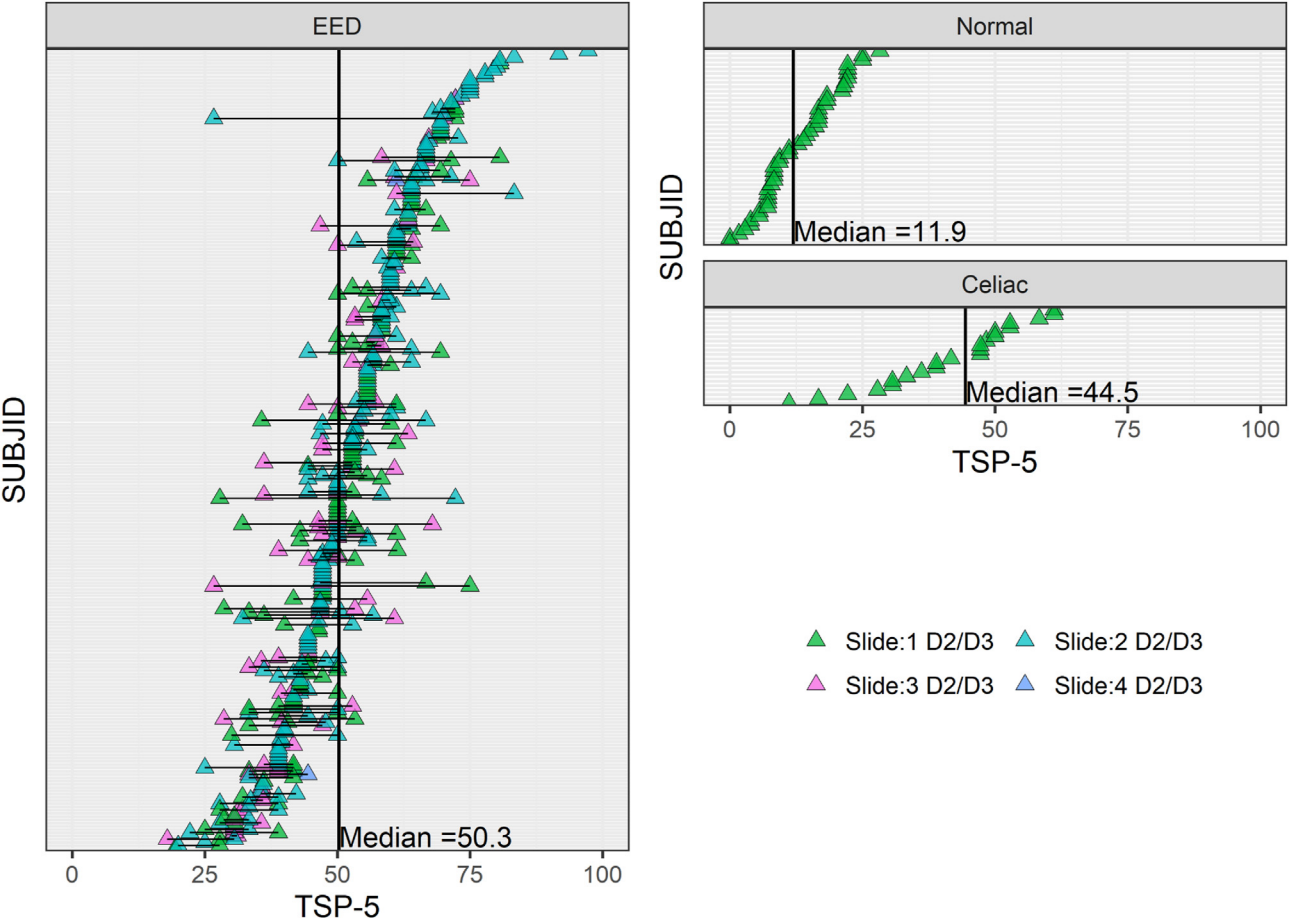
TSP-5 scores in EED biopsies compared with normal and celiac disease biopsies. Biopsies from each child are joined by a horizontal line, and scores are shown ranked in ascending order. Only D2/3 biopsies are shown. Median scores differ slightly from Table 2 as in this figure they are calculated per child, not per biopsy. D2/3 second/third parts of duodenum; EED, environmental enteric dysfunction; SUBJID, participant ID; TSP-5, total score percent for 5 parameters.

**FIGURE 4:**
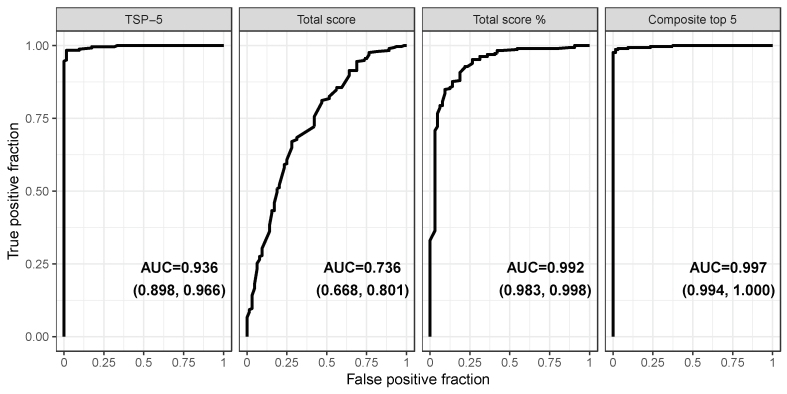
Receiving operating characteristic curves and AUCs showing performance of TSP-5 (left panel) in discriminating between EED and normal biopsies, compared with previous total score (second panel), total score % using 8 parameters (third panel), and composite score derived using imputation for top 5 histology scores (right panel). EED, environmental enteric dysfunction; TSP-5, total score percent for 5 parameters.

The previously published total score and TSP used all 11 individual histology parameters [21]. Total score was a simple sum of scores for the 11 individual parameters. However, the denominator needed to be adjusted for nonscorable values (Table 3). To ensure better comparability across slide images with variable numbers of nonscorable histology parameters, TSP was calculated: the sum of nonmissing individual parameter scores was divided by the total possible score for those combined parameters multiplied by 100 to generate a percentage. To capture this, the coding of intramucosal Brunner’s gland penetration scores was reversed in a revised scoring system so that higher scores represented lesser degrees of Brunner’s gland infiltration (Table 2). This aligned better with the gradient of higher scores indicating greater pathology that is intrinsic to the other histologic parameters. Most of the information required to discriminate between EED and normal biopsies was contained in 5 parameters (IELs, villus architecture, Paneth and goblet cell depletion, and intramucosal Brunner’s glands—the latter reverse-coded) included in the TSP-5; other parameters (Supplemental Figure 1) were less discriminating. TSP-5 scores distinguished normal from EED biopsies (Figure 3). ROC curves comparing AUCs for different index score options showed that the TSP-5 demonstrated an AUC of 0.992 (95% CI: 0.983, 0.998), which was more discriminatory than the original total score (AUC: 0.736; 95% CI: 0.668, 0.805) or the TSP including all 11 histology parameters and presented as a percentage (AUC: 0.936; 95% CI: 0.898, 0.966) (Figure 4).

In view of the number of missing data due to nonscorable parameters (Table 2), a weighted composite score was also derived, using imputation to correct for missing values. This scoring system was only marginally more discriminating than the TSP (AUC: 0.997; 95% CI: 0.994, 1.000; *P* = 0.027) (Figure 4). Confusion matrices showing the results of a cut point between EED and normal identified with training data and applied to test data showed similar results, with the TSP-5 outperforming previous total score iterations and the composite score outperforming TSP-5 in our data (Supplemental Figure 2). However, as TSP-5 can easily be computed arithmetically, and the added discriminatory value was minimal, the composite score was not used in the final analysis.

The highest TSP-5 among the normal biopsies from the North American children was 28.3, and the 95th percentile (conventionally used as an upper limit of normal) was 24.6. Among the 247 children in the EED group with a scorable TSP-5, only 3 (1.2%) and 2 (0.8%) had a score less than the highest and less than the 95th percentile of the comparison group, respectively.

### Regression models

Multivariable regression models (adjusted for technical factors: tissue orientation, staining quality, drying, and crush artifact) were used to test the association between histology features in EED compared with normal and celiac (Table 4). Almost all parameters and the TSP-5 were significantly higher in EED biopsies compared with normal, but Brunner’s gland scores were lower (i.e., fewer in the mucosa), and epithelial detachment did not differ (Table 4). Among the individual histologic parameters, villus architecture, intramucosal Brunner’s glands, and IEL scores showed the highest effect sizes. When comparing EED to celiac disease biopsies, villus architecture, IELs, intramucosal Brunner’s glands, chronic inflammation, and enterocyte injury scores were significantly higher in celiac disease, whereas Paneth and goblet cell depletion were significantly higher in EED compared with celiac biopsies. The TSP-5 was higher in EED than celiac disease (Table 4). Similar regression models were also used to estimate the difference between taking D1 biopsies and D2/3 (Supplemental Table 4).

**TABLE 4:** Generalized estimating equation models of 8 histologic parameters and the total score percent-5 (TSP-5) in EED (reference group), normal, and celiac biopsies, without (univariable) and with (multivariable) adjustment for 3 slide preparation technical parameters.

Term	Univariate analysis			Multivariable analysis		
	Estimate	95% CI	*P*	Estimate	95% CI	*P*
TSP-5						
Intercept^1^	52.6^2^	(51.0, 54.1)	<0.001	62.3^3^	(57.2, 67.4)	<0.001
Normal	−39.7	(−42.4, −37.0)	<0.001	−34.3^4^	(−37.8, −30.8)	<0.001
Celiac	−11.5	(−17.3, −5.6)	<0.001	−5.6	(−11.8, 0.53)	0.07
Villus architecture score						
Intercept	2.30	(2.16, 2.45)	<0.001	2.04	(1.49, 2.59)	<0.001
Normal	−1.88	(−2.08, −1.68)	<0.001	−1.96	(−2.27, −1.65)	<0.001
Celiac	0.83	(0.40, 1.26)	<0.001	0.79	(0.30, 1.28)	0.002
Intraepithelial lymphocyte score						
Intercept	1.43	(1.34, 1.53)	<0.001	1.36	(1.06, 1.66)	<0.001
Normal	−1.05	(−1.20, −0.90)	<0.001	−1.2	(−1.41, −0.98)	<0.001
Celiac	1.11	(0.70, 1.52)	<0.001	0.95	(0.51, 1.40)	<0.001
Goblet cell score						
Intercept	1.50	(1.41, 1.58)	<0.001	2.63	(2.40, 2.86)	<0.001
Normal	−1.24	(−1.37, −1.10)	<0.001	−0.62	(−0.80, −0.45)	<0.001
Celiac	−0.77	(−0.98, −0.56)	<0.001	−0.16	(−0.38, 0.06)	0.16
Paneth cell score						
Intercept	1.73	(1.60, 1.87)	<0.001	2.76	(2.35, 3.17)	<0.001
Normal	−1.43	(−1.60, −1.25)	<0.001	−0.70	(−0.91, −0.49)	<0.001
Celiac	−1.09	(−1.33, −0.84)	<0.001	−0.29	(−0.55,− 0.03)	0.029
Intramucosal Brunner’s gland score						
Intercept	0.40	(0.32, 0.48)	<0.001	0.43	(0.14, 0.71)	0.003
Normal	1.67	(1.32, 2.01)	<0.001	1.64	(1.25,2.03)	<0.001
Celiac	2.26	(1.93, 2.59)	<0.001	2.24	(1.89, 2.59)	<0.001
Chronic inflammation						
Intercept	1.39	(1.33, 1.45)	<0.001	1.29	(1.08, 1.51)	<0.001
Normal	−0.60	(−0.72, −0.47)	<0.001	−0.63	(−0.8, −0.46)	<0.001
Celiac	0.55	(0.37, 0.73)	<0.001	0.53	(0.32, 0.74)	<0.001
Enterocyte injury						
Intercept	0.35	(0.31, 0.39)	<0.001	0.37	(0.23, 0.51)	<0.001
Normal	−0.20	(−0.27, −0.12)	<0.001	−0.21	(−0.31, −0.1)	<0.001
Celiac	0.48	(0.26, 0.69)	<0.001	0.47	(0.24, 0.7)	<0.001
Epithelial detachment						
Intercept	1.0	(0.94, 1.07)	<0.001	1.08	(0.81, 1.35)	<0.001
Normal	−0.09	(−0.24, 0.06)	0.24	−0.02	(−0.22, 0.17)	0.83
Celiac	0	(−0.15, 0.15)	0.97	0.12	(−0.08, 0.31)	0.25

The *P* values shown are derived from generalized estimating equations.

Abbreviations: EED, environmental enteric dysfunction; TSP-5, total score percent-5.

1 The intercept in the univariate models represents the mean value for the histology parameter scores among the EED reference group.

2 The coefficient in the univariate models represents the difference in the histology parameter score or TSP-5 compared with the EED reference group. For example, the TSP-5 is 39.7 (percentage) points lower among the normal cohort compared with the EED cohort and the villus architecture score is 1.88 lower among the normal compared with the EED cohort.

3 The intercept in the multivariable models represents the mean value for the histology parameter score among the EED reference group, if all adjustment covariates (biopsy orientation, staining quality, dry/crush artifact) are zero.

4 The coefficient in the multivariable models represents the difference in the histology parameter score or TSP-5 compared with the EED reference group, adjusting for all other predictors.

### Differences between geographic EED centers

**FIGURE 5:**
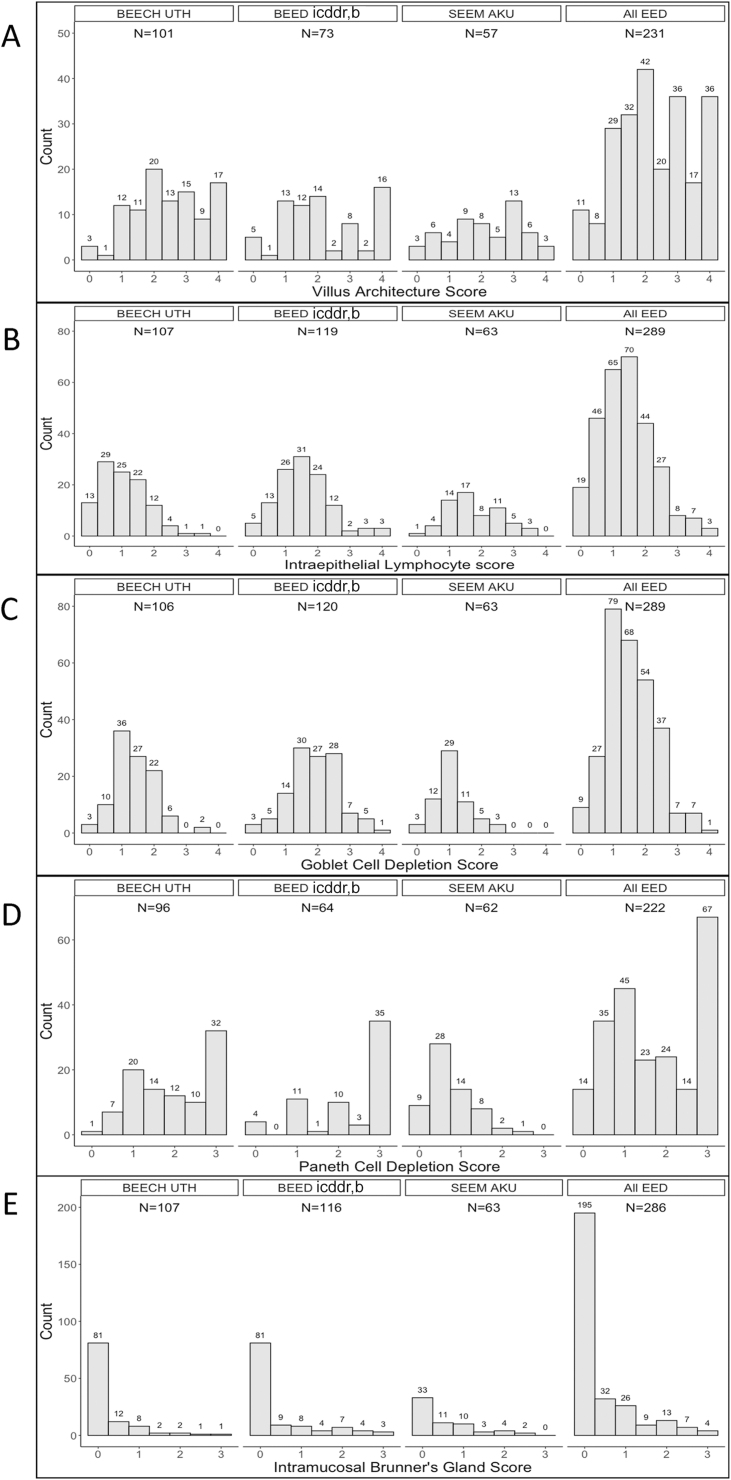
Geographic differences between study centers. Distribution of scores for each parameter are shown for each study center, and the number in each group is shown above each column. (A) Villus architecture score. (B) Intraepithelial lymphocyte score showing a preponderance of higher scores in SEEM. (C) Goblet cell depletion score showing higher scores in BEED. (D) Paneth cell depletion score showing lower scores in SEEM. (E) Intramucosal Brunner’s gland score. AKU, Aga Khan University; BEECH, Biomarkers of Environmental Enteropathy in Children; BEED, Bangladesh Environmental Enteric Dysfunction; EED, environmental enteric dysfunction; icddr,b, International Centre for Diarrhoeal Disease Research, Bangladesh; SEEM, Study of Environmental Enteropathy and Malnutrition; TSP-5, total score percent for 5 parameters; UTH, University Teaching Hospital.

Differences in histology parameter scores between the EED centers were apparent (Table 2 and Figure 2) and analyzed in full (Supplemental Table 5 and Figure 5). In summary, IEL scores were greater in the South Asian centers than in BEECH. Goblet cell depletion scores were greater (i.e., more depleted) in biopsies from BEED than those from other centers, and Paneth cell depletion scores were lower (i.e., not as depleted) in the SEEM biopsies than in those from the other 2 centers. Villus architectural change (blunting) was more severe in BEECH than SEEM. Differences in other parameter scores were not statistically significant between EED sites.

### Intraindividual variation between biopsies among children with EED

Of the 291 children with EED, 132 had >1 slide image from biopsies taken from the second or third part of the duodenum. The intraclass correlation in the TSP-5 was high (0.75) among these biopsies taken from the same individuals on the same day, indicating limited intraindividual variation (FIGURE 2, FIGURE 3).

## Discussion

EED is a global health problem with adverse consequences for the long-term well-being of millions of children living in LMICs. Biopsy material from children is difficult to obtain but can be highly informative [23,24], so we used the opportunity of several parallel, if not identical, studies to make comparisons between EED, normal, and celiac biopsies. The research centers included centers previously reported, one additional EED center [25], and 2 new United States sites. Using a panel of 5 expert gastrointestinal pathologists, masked to biopsy origin, we identified 5 features of this enteropathy that, taken together in a summary index score, the TSP-5, clearly discriminate between EED and biopsies from United States children that were reported as normal. Four of these (villus blunting, intraepithelial lymphocytosis, goblet cell depletion, and Paneth cell depletion), were more frequent in EED; penetration of Brunner’s glands into the mucosa was less frequently observed in EED than in the United States comparison biopsies. We showed that the TSP-5 enhances discrimination over our previously published 11-parameter score in differentiating EED biopsies from those from United States children presenting for endoscopy but without identifiable small bowel disease. TSP-5 has a very high degree of accuracy (AUC values consistently well over 95%), only marginally surpassed by a more complex composite score of the same 5 parameters requiring weighting and imputation of nonscorable parameters. This more complex scoring system was not adopted owing to only marginally improved accuracy and the simplicity and ease of use of TSP-5. The semiquantitative TSP-5 is easily determined by an experienced pathologist, simple to calculate, provides a summative EED severity score, and is most accurate when applied to well oriented, well stained biopsies taken from the second or third part of the duodenum.

Our data show that secretory cell depletion is a characteristic of EED. Paneth cell abnormalities have previously been reported in Crohn’s disease [26] and in Zambian adults living with HIV or nutritional stress [27]. Starvation has been shown to compromise Paneth cells [28]. Goblet cells are well known to be depleted in severe ulcerative colitis [29]. The common depletion of goblet and Paneth cells is of great interest and may be an important characteristic of EED. Evidence of secretory cell dedifferentiation has recently been reported in adults with EED [30]. This distinct feature of EED also adds to the growing literature about the histologic features of various enteropathies that, though at first superficially similar, do differ in subtle but potentially important ways [[31], [32], [33], [34], [35], [36], [37], [38], [39]].

There is genuine difficulty finding appropriate reference biopsies against which to compare EED biopsies. To address this issue, we applied the TSP-5 score to archival biopsy cases from AKU that were obtained from 7 nonmalnourished children in Pakistan. Though considered to have normal mucosal architecture on diagnostic pathology reports, 1 showed *G. intestinalis* trophozoites, and *H. pylori* was observed in 3. *H. pylori* has previously been associated with enteropathy [40]. Detailed scoring clearly demonstrated that the “AKU normal” biopsy scores were much closer to that of EED biopsies than normal biopsies from North America. These results are consistent with work in Zambia and Pakistan, which suggests that, at least in poor communities, EED can be ubiquitous [21,41]. This means that comparison with an outgroup, such as reasonably healthy United States children, is the most appropriate way of defining pathology in a condition such as EED, which is largely geographically determined. Previous studies have used the same approach in analyses of biopsies [42,43], studies of permeability [44], and transcriptomic analysis [45].

There was evidence of significant histologic variation between geographic EED cohorts. This reflects the power of the current study, which, to our knowledge, is the first to attempt direct comparisons of biopsies from children in countries affected or unaffected by EED. More data need to be collected from other settings in Africa, Latin America, the Caribbean, and elsewhere in Asia. We initially postulated that Africa and South Asia might show differences because of prevailing pathogen burden, diet, or other environmental factors, but only the severity of intraepithelial lymphocytosis consistently differed in this manner. The severity of goblet cell depletion was considerably more marked in Bangladesh than the other 2 centers, and biopsies from Pakistan exhibited considerably less Paneth cell depletion than did biopsies from the other 2 centers. Thus, the South Asian centers did not show consistent similarities. The reasons underlying these differences could be of great interest in elucidating etiology. Possible drivers may include variation in pathogen burdens (for example, *H. pylori* and *Giardia* infection [46]) or the microbiota. It is possible, however, that they could reflect differences in inclusion criteria [20]. In the BEED study, children aged between 12 and 18 mo, who were stunted (LAZ < −2) and those who were at risk of stunting (LAZ < −1 to −2) and failed to respond to nutritional therapy underwent endoscopy [25]. In the SEEM study, recruitment was based on nonresponsive wasting [8]. Children in the BEECH study underwent endoscopy on the basis of nonresponsive stunting or wasting, though a minority (*n* = 8) had wasting. However in a companion paper in this supplement, there is also a direct comparison of transcriptomic profiles in the EED centers, which revealed only very modest differences in gene expression between study centers [47]. Future work comparing geographic centers will therefore need greater harmonization of inclusion criteria.

Previous reports have described villus blunting as a prominent feature of EED, but of less severity than observed in celiac disease. Villus blunting has also been confirmed by computer-aided morphometry as described in a companion report in this series [48], and the dense intraepithelial lymphocytosis in EED has been directly compared with celiac disease using immunohistochemistry [49]. In rural Gambian children, compared with age-matched UK controls, chronic cell-mediated enteropathy with crypt hyperplasia, villus blunting, and high densities of IELs were seen regardless of nutritional status [43]. A recent study conducted on Bangladeshi adults showed that malnourished adults (BMI < 18.5 kg/m^2^) with probable EED had significantly higher frequency of subtotal villus atrophy, crypt hyperplasia, and marked cellular infiltration than the controls (BMI > 18.5 kg/m^2^) [50]. In another study comparing duodenal histopathologic features of EED pathology, biopsies of the small bowel from Zambia and Pakistani children showed villus blunting is common, but the intraepithelial lymphocytosis was particularly marked in the Pakistani cohort, and loss of secretory cell lineages was more pronounced in the Zambian cohort [21]. These findings are consistent with the geographic variations that we observed in the current study. In future work, it will be important to include other geographic locations to assess generalizability. Additional features (chronic inflammation, epithelial injury, and epithelial detachment) may provide further mechanistic information. Epithelial injury in particular is emerging as a prominent feature of EED [11]. Although chronic inflammation in the lamina propria did not discriminate well between EED, normal, and celiac biopsies, this does not mean it is unimportant. In another paper in this supplement [49], it was noted that B cell densities appear to have discriminatory potential. Future work may include B cell immunostaining to explore this further.

This study has important limitations. Possibly most important is the age difference between EED cases and United States controls. It is ethically unacceptable to collect endoscopic biopsies without some possibility of benefit to the child [19]. Although we believe that there was a robust justification for biopsies in the children with EED, most biopsies collected in North America were from somewhat older children with abdominal symptoms. Consequently, there was minimal overlap between the ages of the children in EED and control groups, such that collinearity of age and disease variables was inevitable, and age had to be dropped from regression models. The second is that the intensity and color of staining using H&E was less consistent in Bangladesh and Zambia than in the other centers, and no orientation prior to fixation was attempted in Pakistan. There are important batch effects between reagents and differences between suppliers of organic solvents and stains, which may have contributed to variable staining. Hence, although our analysis confirmed accurate discrimination between EED, normal, and celiac biopsies, and differences between geographic centers were still apparent after adjusting for technical variations, the possibility remains that some detail was lost. Lastly, children in Bangladesh and Zambia were screened for parasitic infections prior to endoscopy, but this was not done for the children in Pakistan. In contrast, the strengths of the study include blinding of independent pathologist scoring (with considerable concordance levels between observers, Supplemental Table 6), and a large sample size, which allowed a robust statistical treatment of geographic and technical differences between geographies.

In summary, we present a scoring system suitable for widespread application in research on the histopathology of EED. Five parameters were sufficient to determine the presence of EED with a high degree of precision. Biopsies should be taken from the second or third parts of the duodenum to maximize consistency and reproducibility. We therefore recommend the TSP-5 as a practical way of scoring biopsies from children who may have EED and hope that this will facilitate future rigorous comparisons across populations in relation to pathogen profiles, dietary intakes, and environmental factors. We also reasoned that this scoring system could be applied as an endpoint in clinical trials of novel therapies to reduce adverse consequences of environmentally mediated gut damage.

## Author contributions

The authors’ responsibilities were as follows – CM, PK, TA, MM, SAA, SRM, WAP: designed research; ZJ, CM, MSH, PK, SS, TA, MM, SAA, SRM, WAP: conducted research; DC, KV, SM, GS: analyzed data; TL, CAM, GJT, ÖHY, SSR: generated histology scores; DMD, PBS, PIT: data management; DMD, PBS, PIT, KV, TL, CAM: data interpretation; IMN: telepathology management; ZJ, PK: wrote the paper; DMD, PIT, TL: edited the manuscript; PK: had primary responsibility for final content; and all authors: read and approved the final manuscript.

### Conflict of interest

The authors report no conflicts of interest.

### Funding

The EEDBI Consortium was funded by the following grants: Bill and Melinda Gates Foundation OPP1152812, OPP1066118, OPP1136759, OPP1138727, and OPP1144149, and Advanced Imaging and Tissue Analysis Core of the Washington University Digestive Diseases Research Core Center P30DK052574.

### Data availability

Data described in the manuscript, code book, and analytic code will be made available upon request to the corresponding author pending application and approval.

## Figures and Tables

**Table:** 

**Surname**	**Name**	**Full Affiliation**	**Email**
Ahmed	Kumail	Department of Paediatrics and Child Health, Aga Khan University, Karachi, Pakistan	kumail.ahmed@aku.edu
Ahmed	Sheraz	Department of Paediatrics and Child Health, Aga Khan University, Karachi, Pakistan	sheraz.ahmed@aku.edu
Alam	Md. Ashraful	Nutrition Research Division, International Centre for Diarrhoeal Disease Research, Bangladesh, Dhaka, Bangladesh	mashraful@icddrb.org
Begum	S.M. Khodeza Nahar	Department of Pathology, Bangladesh Specialized Hospital, Dhaka, Bangladesh	khodeza33@hotmail.com
Besa	Ellen	Tropical Gastroenterology & Nutrition Group, University of Zambia School of Medicine, Lusaka, Zambia	ellen@tropgan.net
Chandwe	Kanta	Tropical Gastroenterology & Nutrition Group, University of Zambia School of Medicine, Lusaka, Zambia	kantachandwe@gmail.com
Chipunza	Miyoba	Tropical Gastroenterology & Nutrition Group, University of Zambia School of Medicine, Lusaka, Zambia	miyobachipunza@gmail.com
Das	Subhasish	Nutrition Research Division, International Centre for Diarrhoeal Disease Research, Bangladesh, Dhaka, Bangladesh	subhasish.das@icddrb.org
Denson	Lee A.	Division of Pediatric Gastroenterology, Hepatology, and Nutrition, Cincinnati Children's Hospital Medical Center, Cincinnati, OH, USA	Lee.Denson@cchmc.org
Fahim	Shah Mohammad	Nutrition Research Division, International Centre for Diarrhoeal Disease Research, Bangladesh, Dhaka, Bangladesh	mohammad.fahim@icddrb.org
Gazi	Md. Amran	Nutrition Research Division, International Centre for Diarrhoeal Disease Research, Bangladesh, Dhaka, Bangladesh	amran.gazi@icddrb.org
Hasan	Md. Mehedi	Nutrition Research Division, International Centre for Diarrhoeal Disease Research, Bangladesh, Dhaka, Bangladesh	md.hasan@icddrb.org
Hotwani	Aneeta	Department of Paediatrics and Child Health, Aga Khan University, Karachi, Pakistan	aneeta.hotwani@aku.edu
Iqbal	Junaid	Department of Paediatrics and Child Health, Aga Khan University, Karachi, Pakistan	junaid.iqbal@aku.edu
Iqbal	Najeeha Talat	Department of Paediatrics and Child Health, Aga Khan University, Karachi, Pakistan	najeeha.iqbal@aku.edu
Jakhro	Sadaf	Department of Paediatrics and Child Health, Aga Khan University, Karachi, Pakistan	sadaf.jakhro@aku.edu
Kabir	Furqan	Department of Paediatrics and Child Health, Aga Khan University, Karachi, Pakistan	furqan.kabir@aku.edu
Lawrence	Sarah	Department of Pediatrics, University of Washington School of Medicine, Seattle, WA, USA	Slawrence0008@gmail.com
Mann	Barbara J.	Department of Medicine, University of Virginia School of Medicine, Charlottesville, Virginia, USA	bjm2r@virginia.edu
Mazumder	Ramendra Nath	Nutrition Research Division, International Centre for Diarrhoeal Disease Research, Bangladesh, Dhaka, Bangladesh	ramen@icddrb.org
Memon	Waheeda	Department of Paediatrics and Child Health, Aga Khan University, Karachi, Pakistan	waheeda.memon@aku.edu
Morgan	Brooks	Department of Epidemiology, University of Washington School of Public Health, Seattle, USA	bwmorgan@uw.edu
Mudenda	Victor	Tropical Gastroenterology & Nutrition Group, University of Zambia School of Medicine, Lusaka, Zambia	mudenda2003@yahoo.co.uk
Mulenga	Chola	Tropical Gastroenterology & Nutrition Group, University of Zambia School of Medicine, Lusaka, Zambia	cholamulenga94@gmail.com
Mweetwa	Monica	Tropical Gastroenterology & Nutrition Group, University of Zambia School of Medicine, Lusaka, Zambia	monica@tropgan.net
Qureshi	Abdul Khalique	Department of Paediatrics and Child Health, Aga Khan University, Karachi, Pakistan	abdul.qureshi@aku.edu
Rahman	Masudur	Department of Gastroenterology, Sheikh Russel National Gastroliver Institute and Hospital, Dhaka, Bangladesh	drmasud47@yahoo.com
Rahman	Najeeb	Department of Paediatrics and Child Health, Aga Khan University, Karachi, Pakistan	najeeb.rahman@aku.edu
Sadiq	Kamran	Department of Paediatrics and Child Health, Aga Khan University, Karachi, Pakistan	kamran.sadiq@aku.edu
Sarker	Shafiqul Alam	Nutrition Research Division, International Centre for Diarrhoeal Disease Research, Bangladesh, Dhaka, Bangladesh	sasarker@icddrb.org
Umrani	Fayaz	Department of Paediatrics and Child Health, Aga Khan University, Karachi, Pakistan	fayaz.umrani@aku.edu
Zyambo	Kanekwa	Tropical Gastroenterology & Nutrition Group, University of Zambia School of Medicine, Lusaka, Zambia	kanekwa@tropgan.net
