# Supplementary material for: Histopathology underlying environmental enteric dysfunction in a cohort study of undernourished children in Bangladesh, Pakistan, and Zambia compared with United States children
Source: Am J Clin Nutr. 2024 Sep 17;120(Suppl 1):S15–30. doi: 10.1016/j.ajcnut.2024.02.028 (PMC13168970; doi:10.1016/j.ajcnut.2024.02.028)

**Histopathology underlying environmental enteric dysfunction in a cohort study of undernourished children in Bangladesh, Pakistan, and Zambia compared with US children**

**Kelly et al**

**SUPPLEMENTARY MATERIAL**

**Supplemental Table 1** Previous scoring system (reference 21)

| **Acute (neutrophilic) inflammation** | **0**: Neutrophils absent in the entire specimen or only present in lamina propria without epithelial involvement |
| --- | --- |
|  | **1**: 1-2 foci of epithelial neutrophilic infiltration or crypt microabscesses |
|  | **2**: >2 foci of epithelial neutrophilic infiltration or crypt microabscesses, but <50% of mucosa involved |
|  | **3**: ≥ 50% of mucosa involved by epithelial neutrophilic infiltration |
| **Eosinophil infiltration** | **0:** No increase in eosinophils (highly scattered in lamina propria, no intravillous or intercryptal space with >5 eosinophils) |
|  | **1:** Increased eosinophils (intravillous or intercryptal space with >5 eosinophils) involving < 50% of mucosa, with no eosinophilic crypt microabscesses |
|  | **2:** Increased eosinophils (intravillous or intercryptal space with >5 eosinophils) involving > 50% of mucosa, or up to 1 focus of eosinophilic epithelial infiltration or crypt microabscesses per mucosal fragment |
|  | **3:** >2 foci of eosinophilic epithelial infiltration or crypt microabscesses in any mucosal fragment |
| **Chronic inflammation** | **0:** No qualitative increase in mononuclear inflammatory cells (MIC) in lamina propria. Majority of villus bases contain <3 MIC across, on average. |
|  | **1:** Increased MIC, based on villus base displaying 3-5 MIC across, on average. |
|  | **2:** Increased MIC, based on villus base displaying 6-10 MIC across, on average. |
|  | **3:** Increased MIC, based on villus base displaying >10 lymphocytes on average. |
| **Intraepithelial lymphocytes** | **0:** No areas observed with epithelial/lymphocyte ratio >20% |
|  | **1:** Lymphocyte/epithelial ratio >20%, but <50%, in less than 50% of mucosa |
|  | **2:** Lymphocyte/epithelial ratio >20%, but <50%, in greater than 50% of mucosa |
|  | **3:** Lymphocyte/epithelial ratio >50% in less than 50% of mucosa |
|  | **4:** Lymphocyte/epithelial ratio >50% in greater than 50% of mucosa |
| **Villus architecture** | **0:** Majority of villi are >3 crypt lengths long |
|  | **1:** Villi are < 3 but > 1 crypt length long, with abnormality involving < 50% of mucosa. |
|  | **2:** Villi are < 3 but > 1 crypt length long, with abnormality involving > 50% of mucosa |
|  | **3:** Villi absent, or <1 crypt length long, with abnormality involving < 50% of mucosa |
|  | **4:** Villi absent, or <1 crypt length long, with abnormality involving > 50% of mucosa |
| **Intramucosal Brunner’s glands** | **0:** None observed |
|  | **1:** 1-2 foci, none involving > 5 crypt bases |
|  | **2:** 3-5 foci, none involving > 5 crypt bases |
|  | **3:** > 5 foci, or any area of intramucosal Brunner’s glands involving >5 crypt bases |
| **Foveolar cell metaplasia** | **0:** Not observed |
|  | **1**: 1-2 villus tips involved |
|  | **2:** 3-5 villus tips involved |
|  | **3:** > 5 villus tips involved |
| **Goblet cell density** | **0:** Normal goblet cell density (at least 1 goblet cell per 20 enterocytes) in all evaluable mucosal epithelial layer |
|  | **1:** Decreased goblet cells (<1/20 enterocytes) in 1-25% of evaluable mucosal epithelium |
|  | **2:** Decreased goblet cells (<1/20 enterocytes) in 26-50% of evaluable mucosal epithelium |
|  | **3:** Decreased goblet cells (<1/20 enterocytes) in 51-75% of evaluable mucosal epithelium |
|  | **4:** Decreased goblet cells (<1/20 enterocytes) in 76-100% of evaluable mucosal epithelium |
| **Paneth cell density** | **0:** >5 Paneth cells/ crypt, on average |
|  | **1:** 2-4 Paneth cells/ crypt, on average |
|  | **2:** <2 Paneth cell/crypt, involving <50% of crypt bases |
|  | **3:** <2 Paneth cell/crypt, involving >50% of crypt bases |
| **Enterocyte injury** | **0:** Majority of enterocytes (90%) show tall columnar morphology |
|  | **1:** Enterocytes show low columnar (<2:1 L:W ratio), cuboidal or flat morphology, in < 50% of mucosa |
|  | **2:** Enterocytes show low columnar (<2:1 L:W ratio), cuboidal or flat morphology, in > 50% of mucosa |
|  | **3:** Any area of mucosal erosion/ulceration |
| **Epithelial detachment** | **0**: Complete coverage of mucosal surface by epithelial cells |
|  | **1**: Surface epithelium missing or detached from <25% of mucosa |
|  | **2**: Surface epithelium missing or detached from 25-50% of mucosa |
|  | **3**: Surface epithelium missing or detached from 51-75% of mucosa |
|  | **4**: Surface epithelium missing or detached from >75% of mucosa |

**Supplemental Table 2** Distribution of number of slides by Center and by pathologist^1^

| **D2/D3 biopsies** | | | |
| --- | --- | --- | --- |
| **STUDY/ (N Participants, N Slides)** | **N of Slide(s) (N of Participants)** | **N slides scored by 2 pathologists** | **N slides scored by 3 pathologists** |
| Cincincatti Children’s Hospital Medical Center  (48 participants, 48 slides) | 1 (48) | 29 | 19 |
| University of Virginia  (18 participants, 18 slides) | 1 (18) | 11 | 7 |
| Aga Khan University  (63 participants, 180 slides) | 1 (1) | 1 | 0 |
|  | 2 (7) | 12 | 2 |
|  | 3 (55) | 129 | 36 |
| International Centre for Diarrhoeal Disease Research, Bangladesh  (120 participants, 120 slides) | 1 (120) | 92 | 28 |
| University Teaching Hospital (108 participants, 205 slides) | 1 (38) | 38 | 0 |
|  | 2 (44) | 88 | 0 |
|  | 3 (25) | 75 | 0 |
|  | 4 (1) | 4 | 0 |
| **D1 biopsies** | | | |
| Aga Khan University  (54 participants, 54 slides) | 54 (54) | 54 | 0 |
| International Centre for Diarrhoeal Disease Research, Bangladesh  (120 participants, 120 slides) | 120 (120) | 92 | 28 |

^1^In addition to the EED and US cohorts, 7 Aga Khan University archival slides from 7 individuals were scored. Their duodenal location was not specified. All 7 slides were scored by two pathologists.

**Supplemental Table 3 Technical features scoring system**

| **Quality of histology (fixation, sectioning, and/or staining)** | 0: Quality issues, much histopathology uninterpretable |
| --- | --- |
|  | 1: Quality issues, some aspects not readable |
|  | 2: Acceptable quality |
|  | 3: Excellent quality |
| **Tissue Orientation** | 0: All or most tissue is tangentially sectioned precluding definitive assessment of mucosal architecture |
|  | 1: Minority of mucosa (<50%) is longitudinally sectioned |
|  | 2: Majority but not all of mucosa is longitudinally sectioned |
|  | 3: All or almost all mucosa longitudinally sectioned for architectural assessment |
| **Drying, crush, or other artifact** | P: Present |
|  | A: Absent |
| **Pathogens** | Y: Yes (specify pathogen) |
|  | N: No |
|  | U: Uncertain |
| **Number of tissue fragments** |  |

**Supplemental Table 4** Estimates of histologic score differences between D1 biopsies compared to D2/3 biopsies, derived from GEE models

| Histology measurement | Estimate (unadjusted) | 95% CI (unadjusted) | Estimate (adjusted) | 95% CI (adjusted) |
| --- | --- | --- | --- | --- |
| TSP-5 | -9.47 | (-12.11, -6.83) | -10.53 | (-13.18, -7.89) |
| Villus architecture | -0.04 | (-0.37, 0.29) | -0.06 | (-0.41, 0.29) |
| Chronic inflammation | -0.02 | (-0.12, 0.07) | -0.02 | (-0.12, 0.08) |
| Acute inflammation | 0.01 | (-0.01, 0.03) | 0.01 | (-0.01, 0.03) |
| Intraepithelial lymphocytosis | -0.55 | (-0.68, -0.41) | -0.53 | (-0.67, -0.4) |
| Intramucosal Brunner’s glands | 1.23 | (1.02, 1.44) | 1.20 | (0.98, 1.41) |
| Foveolar metaplasia | 0.01 | (-0.02, 0.04) | 0.01 | (-0.02, 0.05) |
| Goblet cell depletion | 0.08 | (-0.03, 0.18) | 0.03 | (-0.08, 0.13) |
| Paneth cell depletion | 0.18 | (0, 0.37) | 0.14 | (-0.05, 0.33) |

Multivariable models adjust for center and age. Estimate refers to difference in histology measurement (with 95%CI) obtained in D1 biopsies compared to D2/D3 (reference)

**Supplemental Table 5 Generalized estimating equation models of eight histological parameters and the total score percent-5 (TSP-5) in the AKU (reference group), icddr,b, and UTH cohorts, without (univariable) and with (multivariable) adjustment for three slide preparation technical parameters.**

|  | **Univariate analysis** | | | **Multivariable analysis** | | | |
| --- | --- | --- | --- | --- | --- | --- | --- |
| **Term** | **Estimate** | **95% CI** | **P** | **Estimate** | **95% CI** | | **p** |
| **TSP-5** | | | | | | | |
| Intercept^1^ | 45.6^2^ | (42.9, 48.2) | <0.001 | 47.4^3^ | (38.5, 56.7) | <0.001 | |
| icddr,b: ref=AKU | 12.9 | (8.9, 16.8) | <0.001 | 12.2 | (7.0, 17.5) | <0.001 | |
| UTH:ref=  AKU | 8.0 | (4.7, 11.2) | <0.001 | 7.5 | (3.4, 11.9) | <0.001 | |
| UTH:ref=  icddr,b | -4.9 | (-8.5, -1.4) | 0.007 | -4.7 | (-8.5, -0.9) | 0.016 | |
| **Villus architecture score** | | | | | | | |
| Intercept | 2.15 | (1.87, 2.42) | <0.001 | 1.44 | (0,.54, 2.34) | 0.002 | |
| icddr,b: ref=AKU | 0.04 | (-0.36, 0.44) | 0.844 | 0.29 | (-0.22, 0.8) | 0.270 | |
| UTH:ref=  AKU | 0.32 | (-0.02, 0.67) | 0.065 | 0.49 | (0.05, 0.93) | 0.029 | |
| UTH:ref=  icddr,b | 0.28 | (-0.07, 0.63) | 0.114 | 0.20 | (-0.19, 0.59) | 0.305 | |
| **Intraepithelial lymphocyte score** | | | | | | | |
| Intercept | 1.77 | (1.57, 1.97) | <0.001 | 1.70 | (1.19,2.21) | <0.001 | |
| icddr,b: ref=AKU | -0.22 | (-0.47, -0.03) | 0.087 | -0.16 | (-0.47,0.15) | 0.302 | |
| UTH:ref=  AKU | -0.67 | (-0.91, -0.43) | <0.001 | -0.59 | (-0.88,-0.3) | <0.001 | |
| UTH:ref=  icddr,b | -0.45 | (-0.65, -0.25) | <0.001 | -0.43 | (-0.64, -0.21) | <0.001 | |
| **Goblet cell score** | | | | | | | |
| Intercept | 1.10 | (0.96, 1.25) | <0.001 | 2.15 | (1.75, 2.54) | <0.001 | |
| icddr,b: ref=AKU | 0.78 | (0.58, 0.98) | <0.001 | 0.46 | (0.21, 0.71) | <0.001 | |
| UTH:ref=  AKU | 0.33 | (0.15, 0.52) | <0.001 | 0.16 | (-0.06, 0.39) | 0.159 | |
| UTH:ref=  icddr,b | -0.45 | (-0.63, -0.27) | <0.001 | -0.30 | (-0.49,-0.1) | 0.002 | |
| **Paneth cell score** | | | | | | | |
| Intercept | 0.74 | (0.62, 0.87) | <0.001 | 1.12 | (0.5, 1.75) | <0.001 | |
| icddr,b: ref=AKU | 1.52 | (1.26, 1.79) | <0.001 | 1.28 | (0.92, 1.64) | <0.001 | |
| UTH:ref=  AKU | 1.27 | (1.06, 1.48) | <0.001 | 1.00 | (0.69, 1.31) | <0.001 | |
| UTH:ref=  icddr,b | -0.25 | (-0.54, 0.04 | 0.088 | -0.28 | (-0.59, 0.03 | 0.075 | |
| **Intramucosal Brunner’s gland score** | | | | | | | |
| Intercept | 0.52 | (0.35, 0.69) | <0.001 | 0.64 | (0.22, 1.06) | 0.003 | |
| icddr,b: ref=AKU | -0.07 | (-0.29, 0.15) | 0.535 | -0.12 | (-0.38,0.13) | 0.348 | |
| UTH:ref=  AKU | -0.25 | (-0.44, -0.05) | 0.012 | -0.32 | (-0.56, -0.08) | 0.008 | |
| UTH:ref=  icddr,b | -0.18 | (-0.35, 0) | 0.053 | -0.20 | (-0.39, 0) | 0.048 | |
| **Chronic inflammation score** | | | | | | | |
| Intercept | 1.31 | (1.21, 1.42) | <0.001 | 1.14 | (0.82, 1.46) | <0.001 | |
| icddr,b: ref=AKU | -0.02 | (-0.17, 0.12) | 0.744 | 0.02 | (-0.16, 0.21) | 0.813 | |
| UTH:ref=  AKU | 0.21 | (0.06, 0.35) | 0.006 | 0.21 | (0.02, 0.39) | 0.028 | |
| UTH:ref=  icddr,b | 0.23 | (0.08, 0.38) | 0.0002 | 0.18 | (0.04, 0.33) | 0.016 | |
| **Enterocyte injury score** | | | | | | | |
| Intercept | 0.40 | (0.32, 0.47) | <0.001 | 0.47 | (0.25, 0.7) | <0.001 | |
| icddr,b: ref=AKU | -0.05 | (-0.16, 0.05) | 0.327 | -0.08 | (-0.21, 0.05) | 0.207 | |
| UTH:ref=  AKU | -0.07 | (-0.18, 0.03) | 0.151 | -0.12 | (-0.23, 0) | 0.052 | |
| UTH:ref=  icddr,b | -0.02 | (-0.12, 0.07) | 0.645 | -0.03 | (-0.13, 0.07) | 0.523 | |
| **Epithelial detachment score** | | | | | | | |
| Intercept | 1.0 | (0.89, 1.11) | <0.001 | 1.07 | (0.66, 1.48) | <0.001 | |
| icddr,b: ref=AKU | 0.05 | (-0.12, 0.22) | 0.585 | 0.00 | (-0.21, 0.21) | 0.983 | |
| UTH:ref=  AKU | -0.03 | (-0.18, 0.12) | 0.674 | -0.07 | (-0.24, 0.11) | 0.459 | |
| UTH:ref=  icddr,b | -0.08 | (-0.25, 0.09) | 0.356 | -0.06 | (-0.25, 0.12) | 0.495 | |

P values shown are derived from Generalized Estimating Equations.

^1^The intercept in the univariate models represents the mean value for the histology parameter scores among the AKU cohort. The intercept in the multivariable models represents the mean value for the histology parameter scores among the AKU cohort if all covariates (biopsy orientation, staining quality, dry/crush artifact) are set to zero (for continuous variables) or reference level (for categorical variables).

^2^The coefficient in the univariate models represents the difference in the histology parameter score or TSP-5 compared to the reference cohort. For example, the TSP-5 is 12.9 (percentage) points higher among the icddr,b cohort compared to the AKU cohort and the villus architecture parameter has a 0.32 higher score among the UTH cohort compared to the AKU cohort.

^3^ The coefficient in the multivariable models represents the difference in the histology parameter score or TSP-5 compared to the reference group, holding all covariates constant.

**Supplemental Table 6** Inter-observer and intra-observer variation

A. Inter-observer agreement calculated on a subset of 60 slides scored by 2 different pathologists

| **Histologic scoring parameter** | **Percent agreement** | **Gwet's AC*** |
| --- | --- | --- |
| Acute inflammation | 99.5 | 0.995 |
| Eosinophilic infiltration | 96.1 | 0.96 |
| Chronic inflammation | 78.7 | 0.58 |
| Intraepithelial lymphocytes | 73.1 | 0.41 |
| Villous architecture | 79.2 | 0.49 |
| Intramucosal Brunner’s glands | 88.9 | 0.78 |
| Foveolar cell metaplasia | 97.8 | 0.98 |
| Goblet cell density | 75.5 | 0.50 |
| Paneth cell density | 76.9 | 0.51 |
| Enterocyte injury | 83.1 | 0.74 |
| Epithelial detachment | 85.4 | 0.74 |

* agreement coefficient

B. Intra-observer agreement across histologic parameters and total score for 35 slides re-scored at different times by the same pathologist

| **Pathologist** | **Number of dual reads** | **Percent agreement** | **Kappa** | **Gwet’s AC*** |
| --- | --- | --- | --- | --- |
| 1 | 15 | 93.9 | 0.63 | 0.87 |
| 2 | 20 | 94.6 | 0.62 | 0.90 |

* agreement coefficient

**Supplemental Figure 1** Parameters not included in the TSP-5 score in EED, normal and celiac biopsies, and compared across geographical centers.

A Epithelial detachment


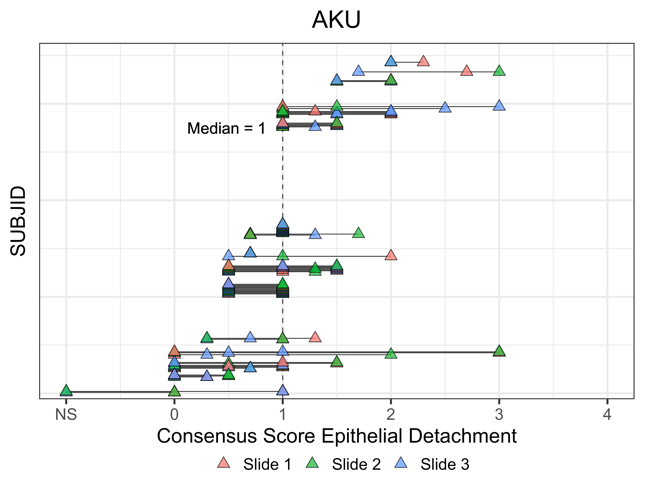

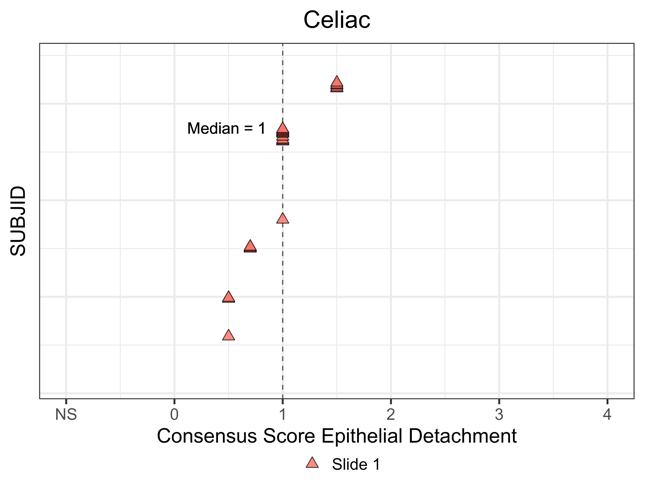

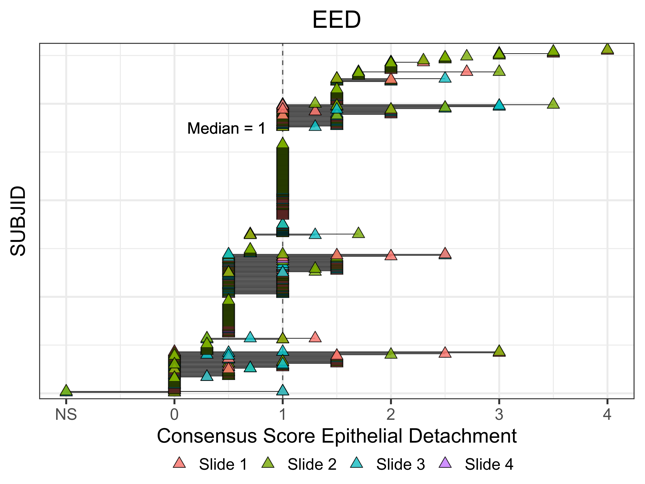

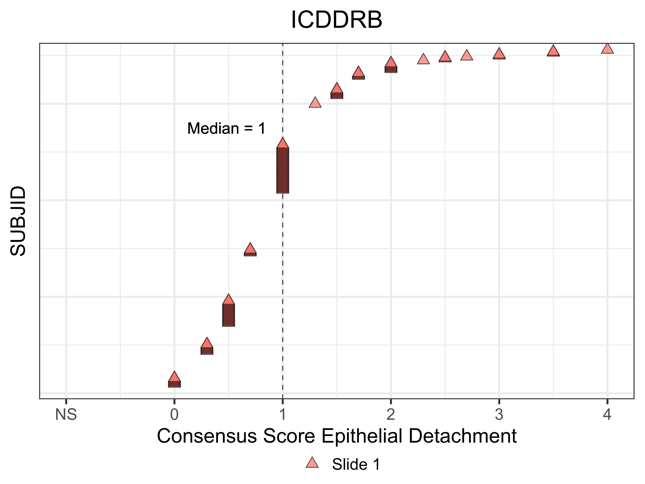

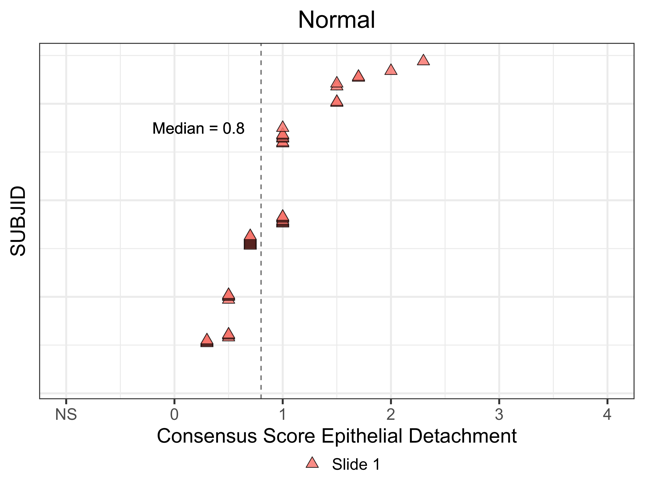

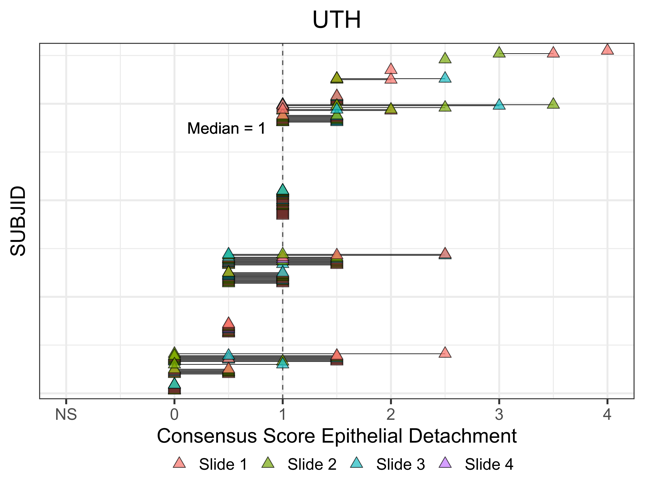


| **Term** | **Estimate^2^** | **95% CI** | **p** |
| --- | --- | --- | --- |
| Intercept^1^ | 1 | (0.72, 1.29) | <0.001 |
| Normal:ref=EED | -0.26 | (-0.5, -0.03) | 0.026 |
| Celiac:ref=EED | -0.12 | (-0.39, 0.15) | 0.39 |
| Age (years) | 0.04 | (0.01, 0.07) | 0.017 |
| TSORIEN | -0.13 | (-0.21, -0.05) | 0.002 |
| HISQUAL | 0 | (-0.14, 0.15) | 0.982 |
| DRCRART | 0.28 | (0.06, 0.51) | 0.013 |

^1^The intercept in these multivariable models represents the mean value for epithelial detachment scores among the EED cohort if all covariates (biopsy orientation, staining quality, dry/crush artifact) are set to zero (for continuous variables) or reference level (for categorical variables).

^2^ The coefficients shown for Normal and Celiac biopsies represent the difference in the histology parameter score or TSP-5 compared to the reference group, holding all covariates constant.

Abbreviations: EED, environmental enteric dysfunction; TSORIENT, tissue orientation score; HISQUAL, histological quality (Mainly determined by staining quality) score; DRCART, drying and crush artifact.

B Enterocyte injury


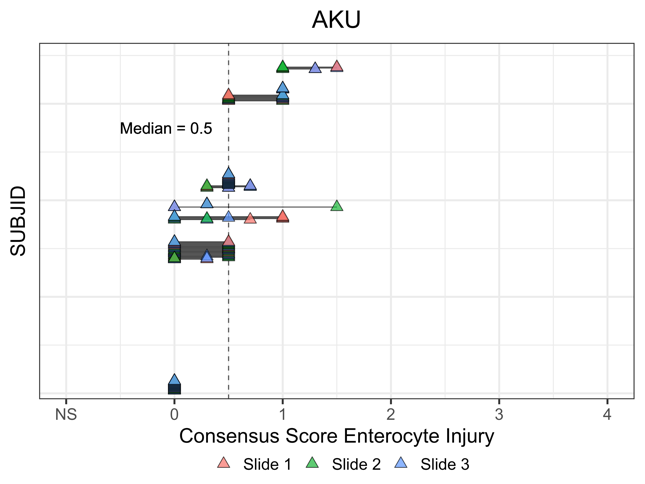

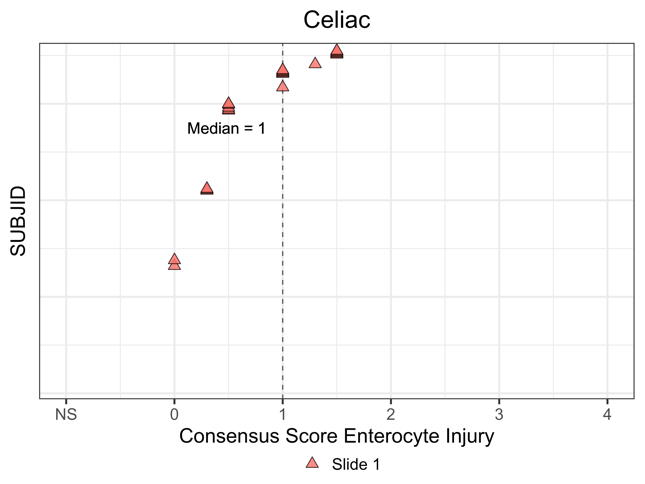

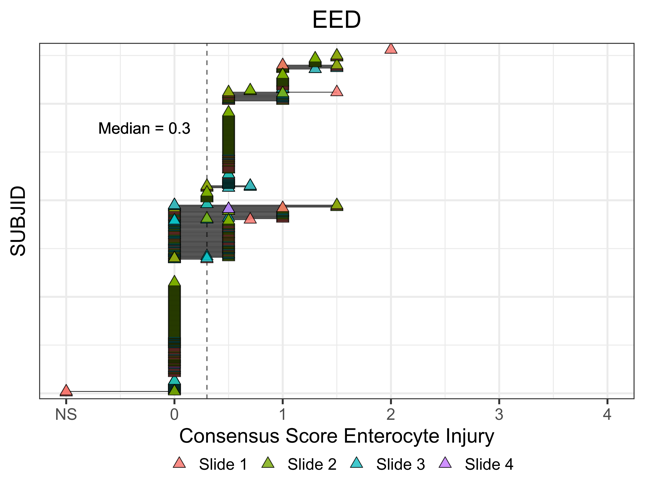

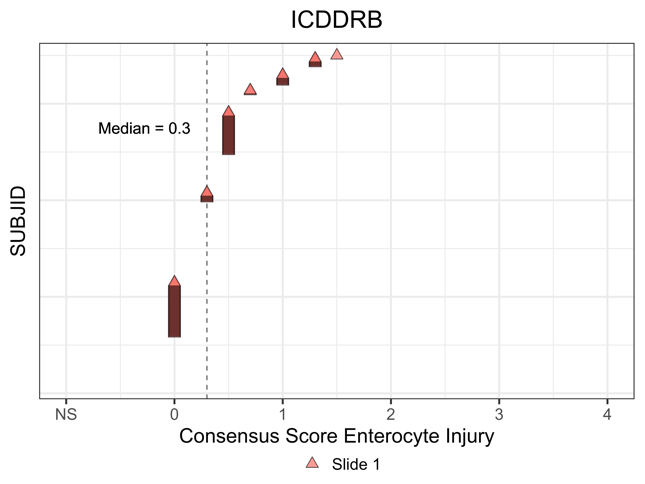

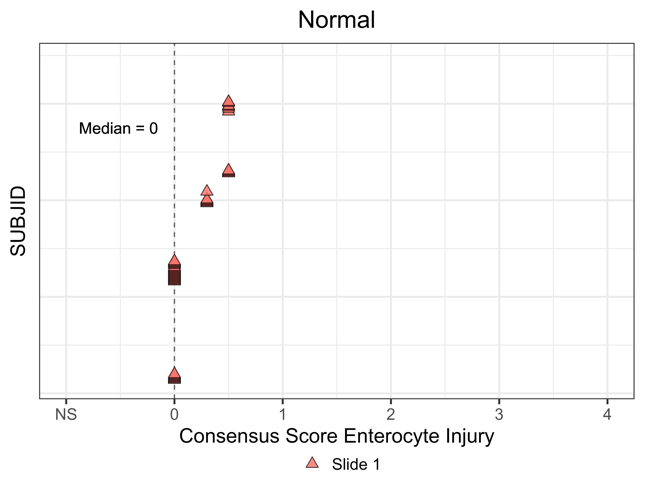

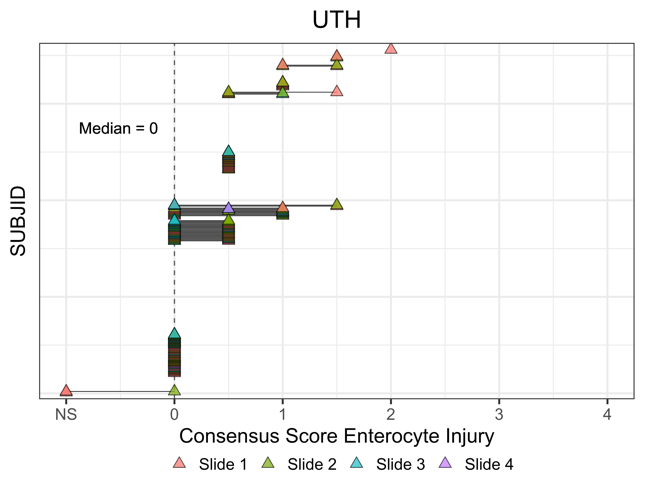


| **Term** | **Estimate^2^** | **95% CI** | **p** |
| --- | --- | --- | --- |
| Intercept^1^ | 0.37 | (0.22, 0.52) | <0.001 |
| Normal:ref=EED | -0.21 | (-0.37, -0.05) | 0.012 |
| Celiac:ref=EED | 0.47 | (0.21, 0.72) | <0.001 |
| Age (years) | 0 | (-0.02, 0.02) | 0.989 |
| TSORIEN | 0.02 | (-0.03, 0.07) | 0.434 |
| HISQUAL | -0.01 | (-0.09, 0.07) | 0.753 |
| DRCRART | -0.07 | (-0.2, 0.05) | 0.25 |

^1^The intercept in these multivariable models represents the mean value for enterocyte injury scores among the EED cohort if all covariates (biopsy orientation, staining quality, dry/crush artifact) are set to zero (for continuous variables) or reference level (for categorical variables).

^2^ The coefficients shown for Normal and Celiac biopsies represent the difference in the histology parameter score or TSP-5 compared to the reference group, holding all covariates constant.

Abbreviations: EED, environmental enteric dysfunction; TSORIENT, tissue orientation score; HISQUAL, histological quality (Mainly determined by staining quality) score; DRCART, drying and crush artifact.

C Chronic inflammation


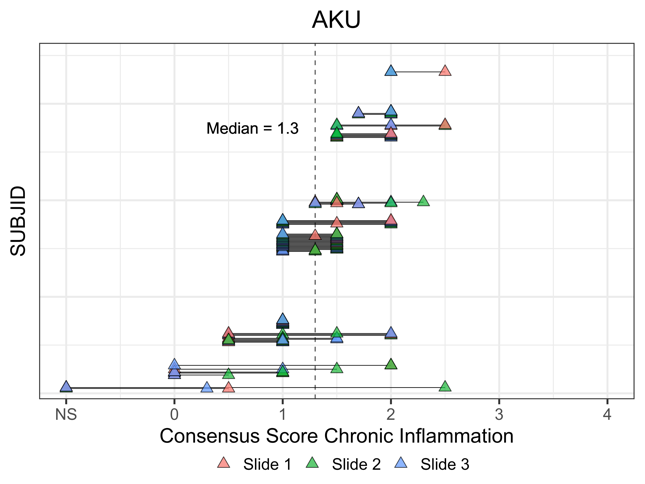

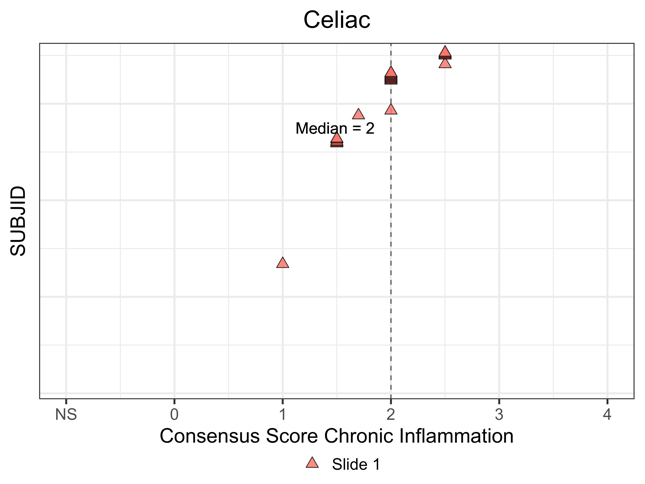

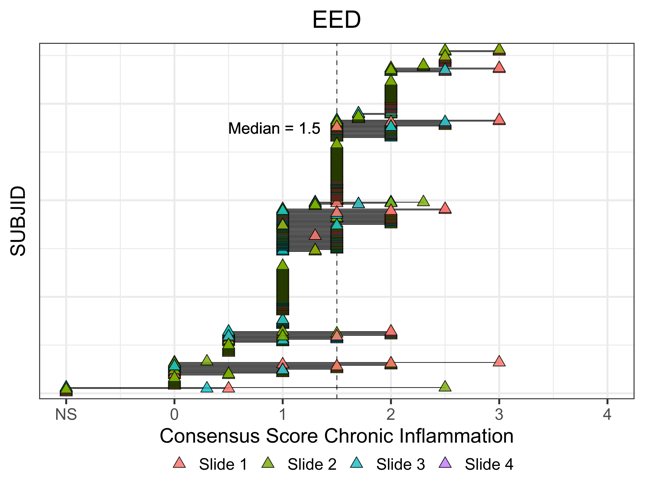

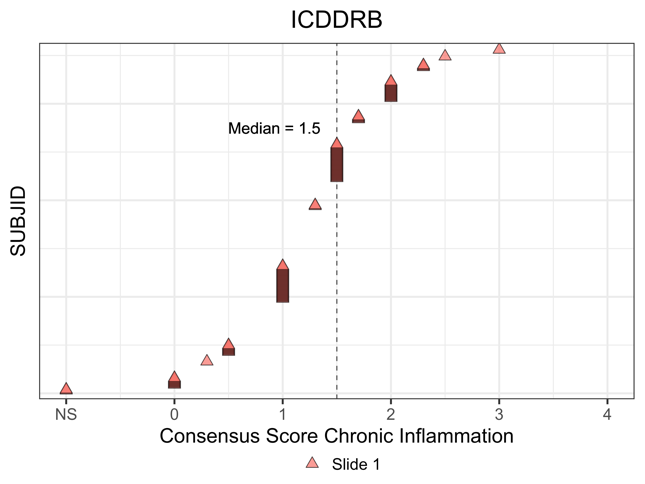

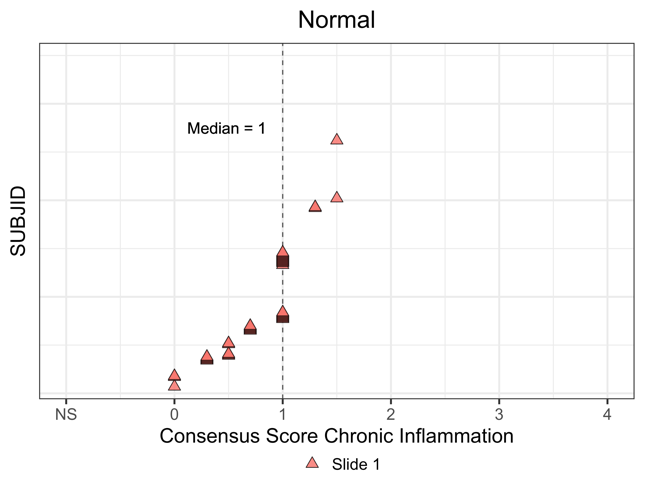

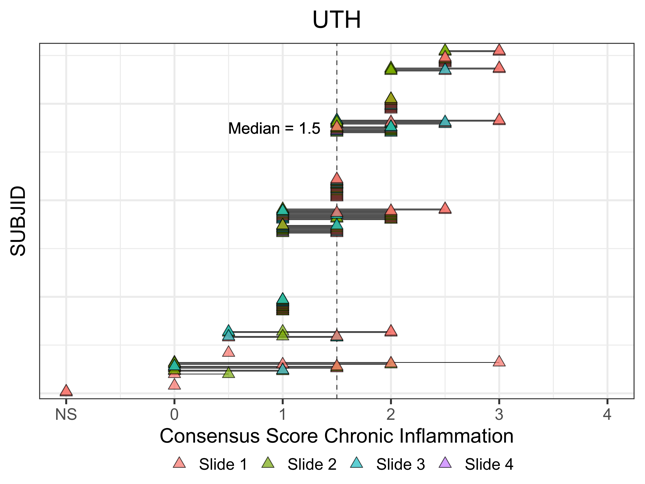


| **Term** | **Estimate^2^** | **95% CI** | **p** |
| --- | --- | --- | --- |
| Intercept^1^ | 1.26 | (1.04, 1.48) | <0.001 |
| Normal:ref=EED | -0.73 | (-0.98, -0.48) | <0.001 |
| Celiac:ref=EED | 0.44 | (0.18, 0.7) | <0.001 |
| Age (years) | 0.01 | (-0.01, 0.04) | 0.179 |
| TSORIEN | 0.08 | (0.01, 0.14) | 0.022 |
| HISQUAL | 0 | (-0.11, 0.11) | 0.997 |
| DRCRART | 0.1 | (-0.11, 0.31) | 0.362 |

^1^The intercept in these multivariable models represents the mean value for chronic inflammation scores among the EED cohort if all covariates (biopsy orientation, staining quality, dry/crush artifact) are set to zero (for continuous variables) or reference level (for categorical variables).

^2^ The coefficients shown for Normal and Celiac biopsies represent the difference in the histology parameter score or TSP-5 compared to the reference group, holding all covariates constant.

Abbreviations: EED, environmental enteric dysfunction; TSORIENT, tissue orientation score; HISQUAL, histological quality (Mainly determined by staining quality) score; DRCART, drying and crush artifact.

**Supplemental Figure 2** Confusion matrices showing predictive power of (A) TSP-5, (B) TSP, (C) Total score, and (D) Composite top 5. Confusion matrices were constructed by partitioning 80% of the data into a training set and defining a cut point using logistic regression to differentiate between EED and normal. The remaining 20% of the data are then used to test the accuracy of the cut point and results are shown below.

**Supplemental Atlas** Atlas of histological appearances


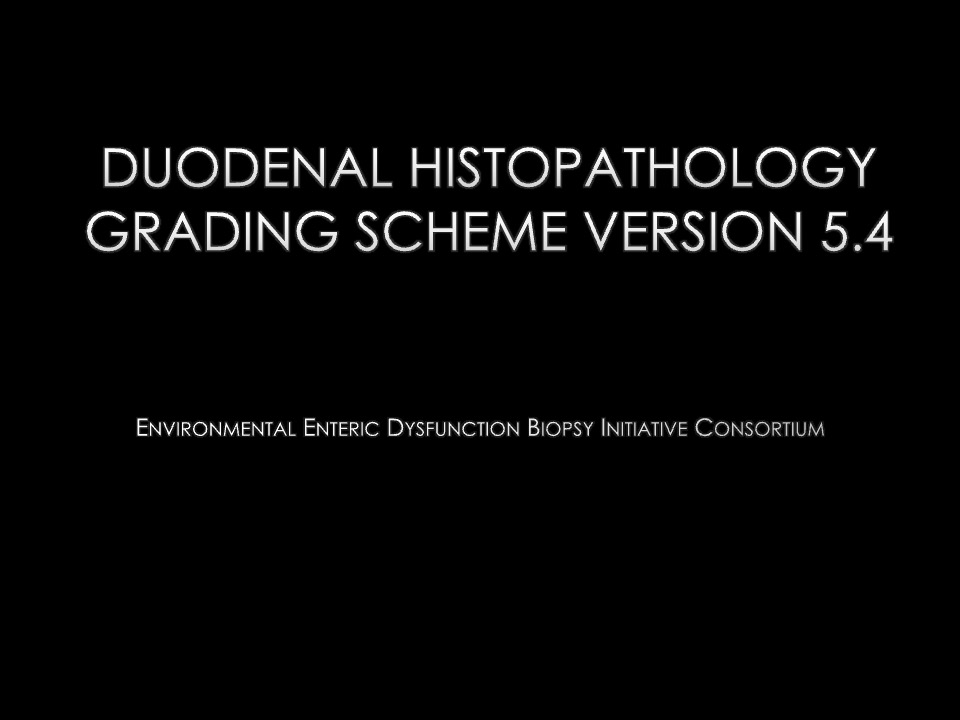

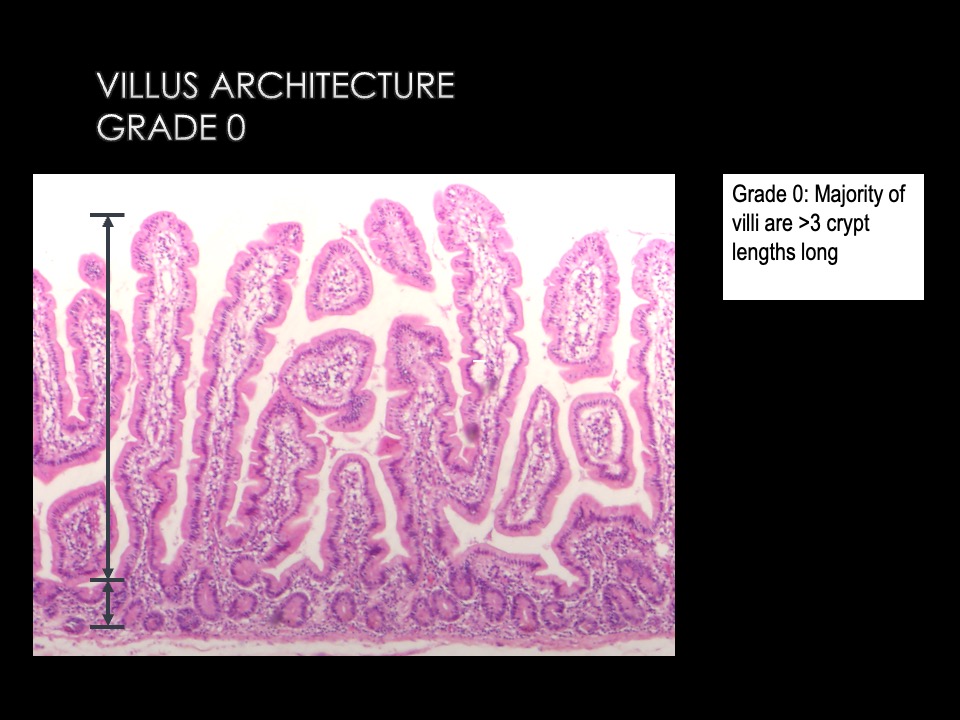

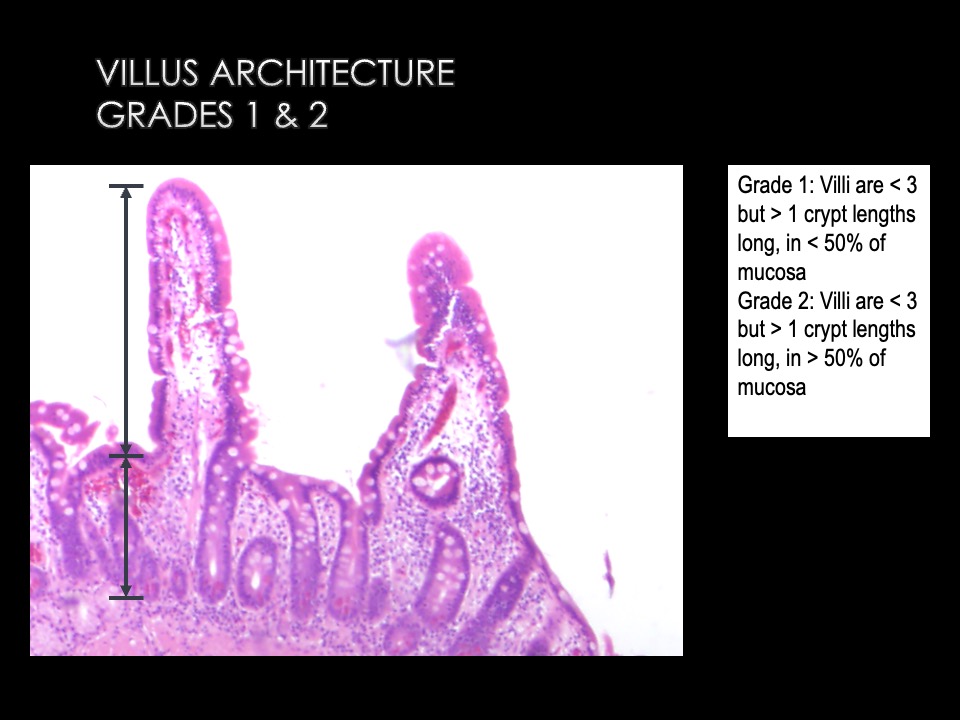

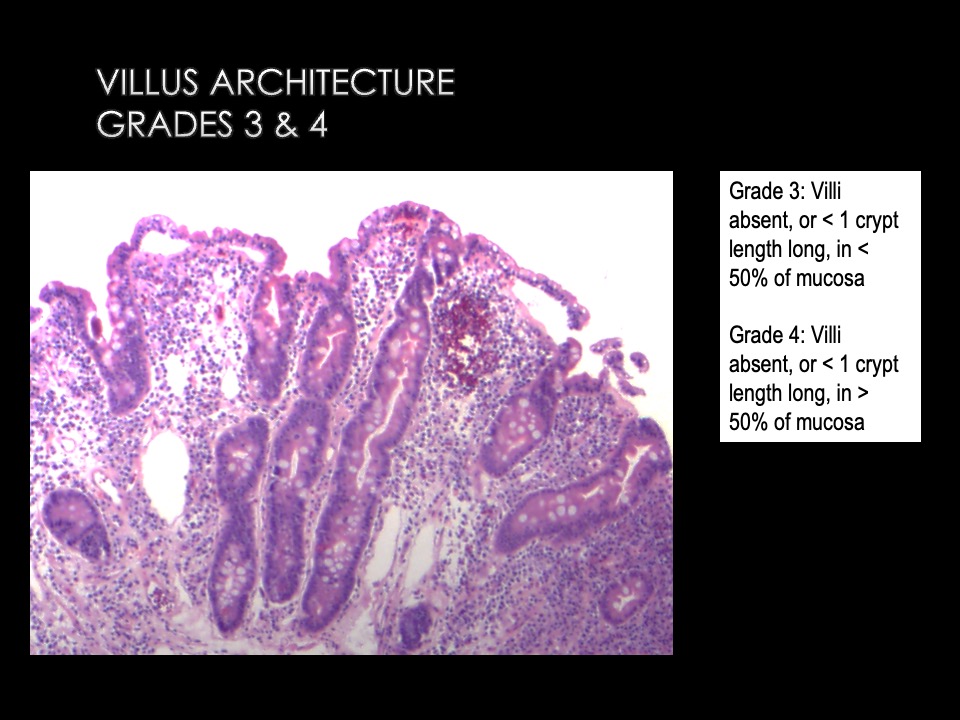

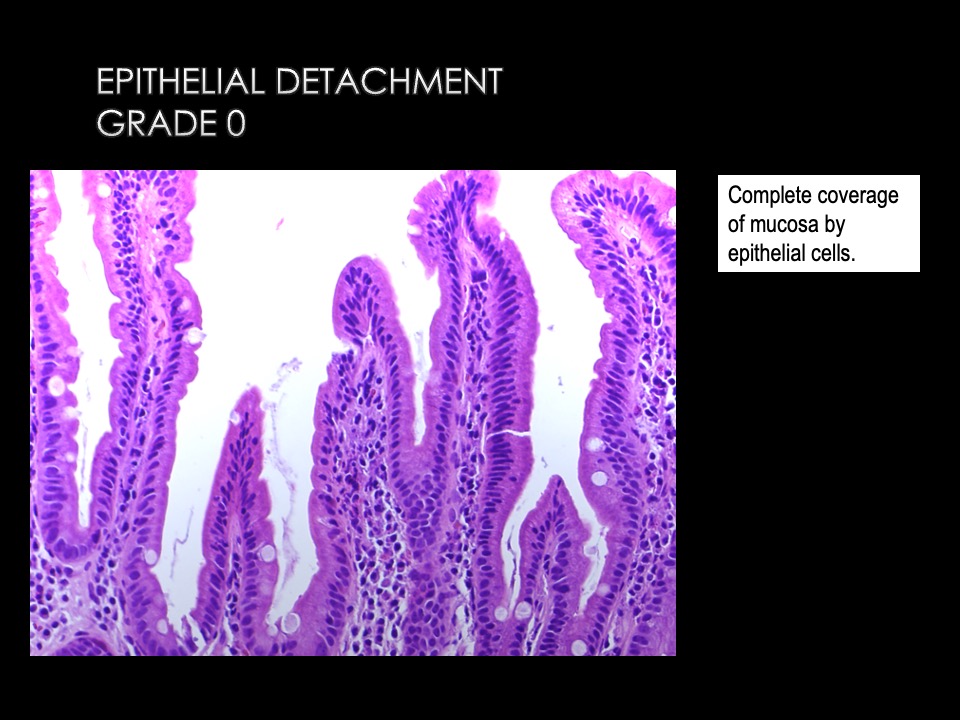

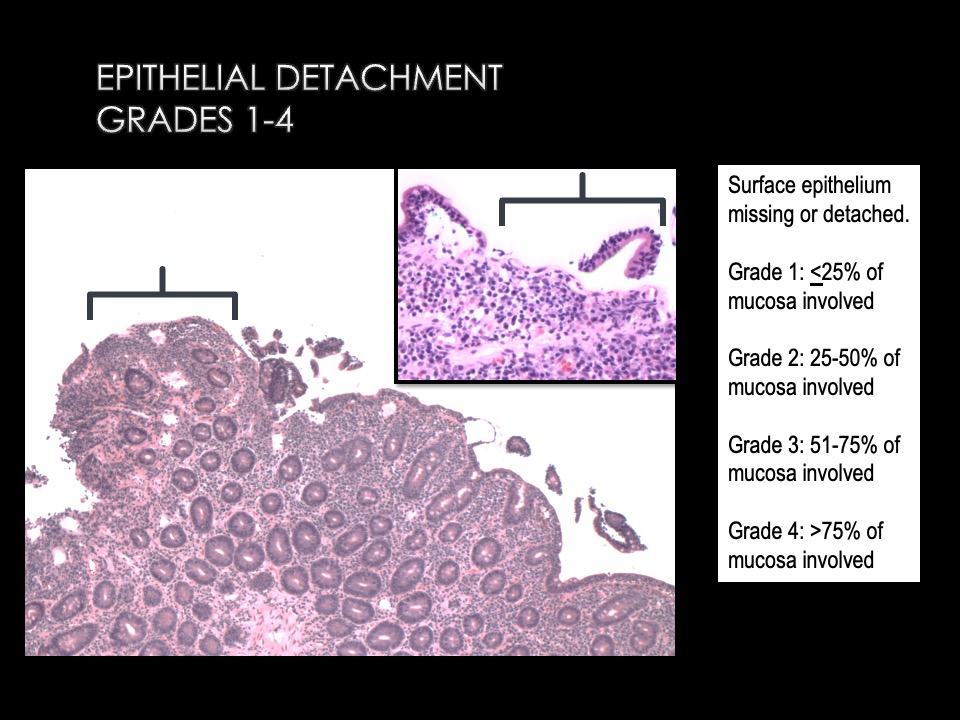

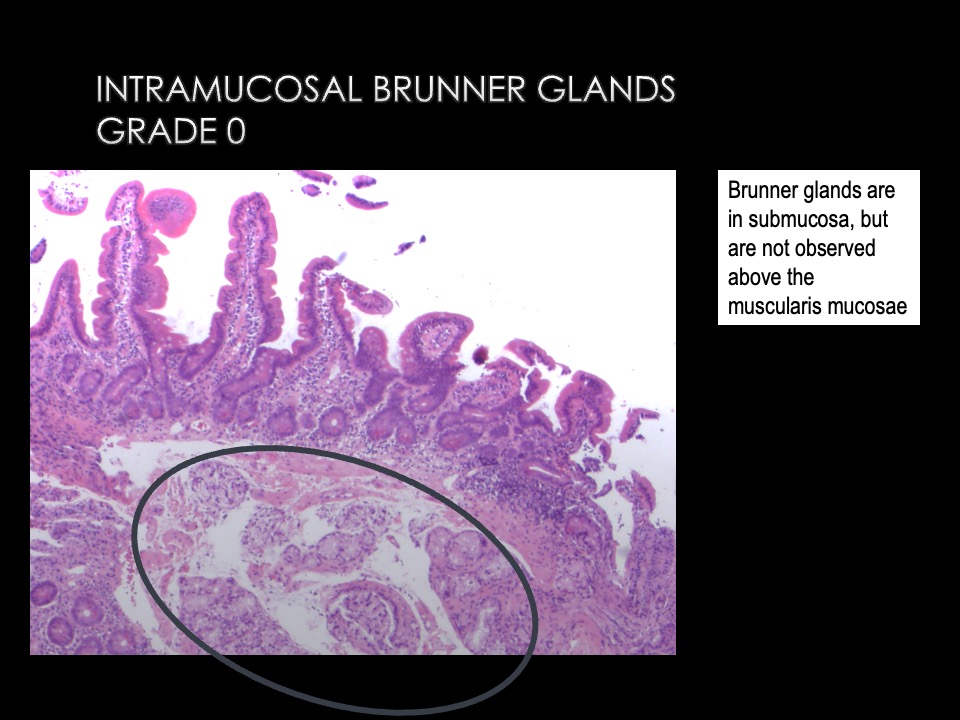

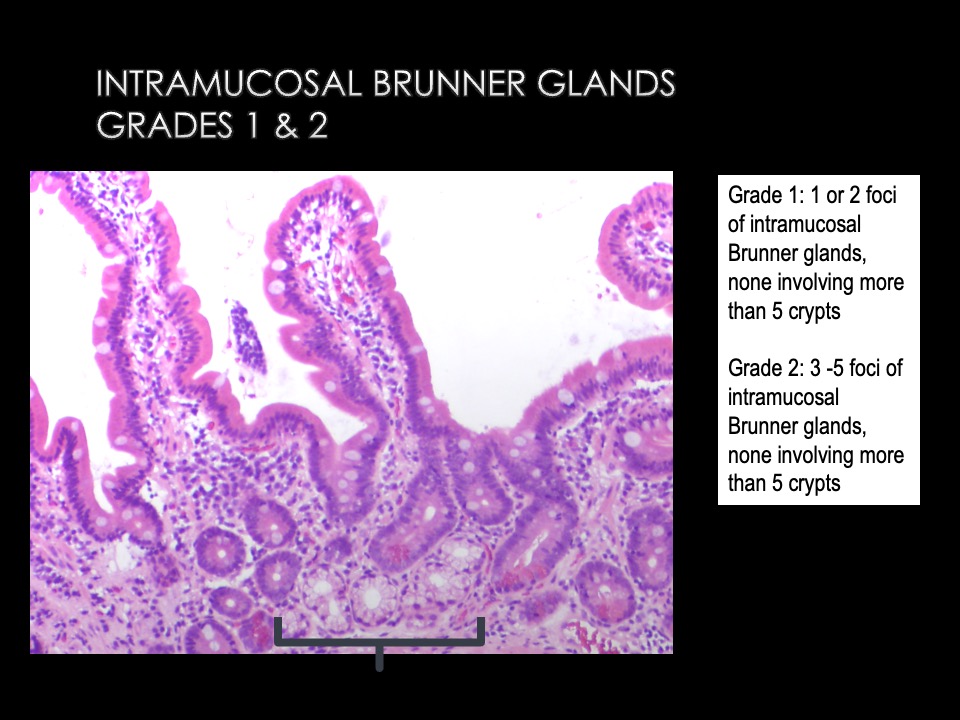

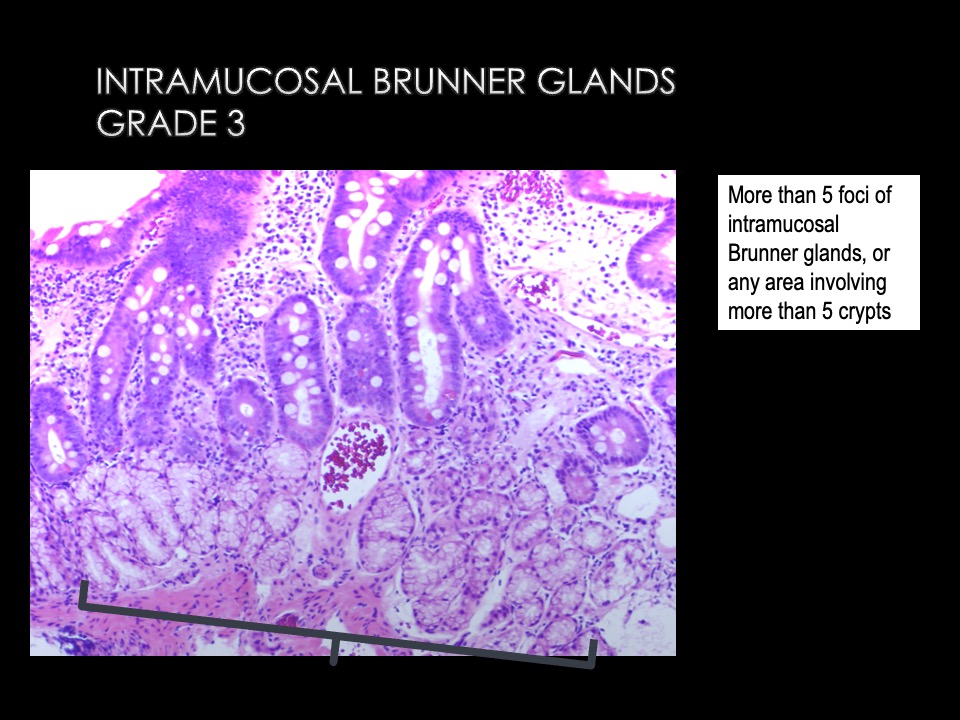

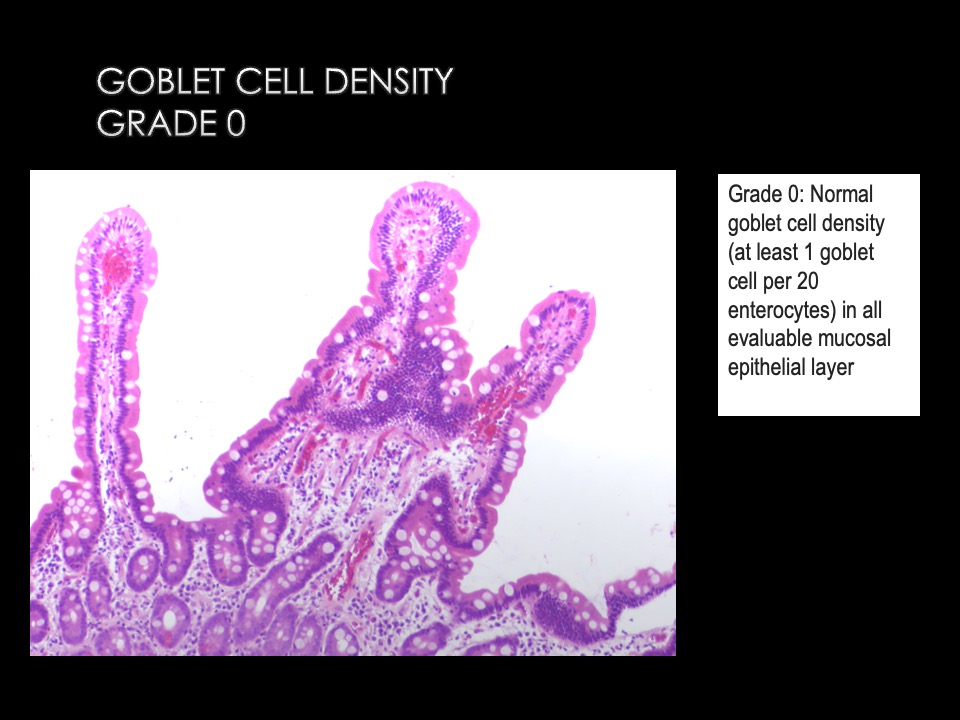

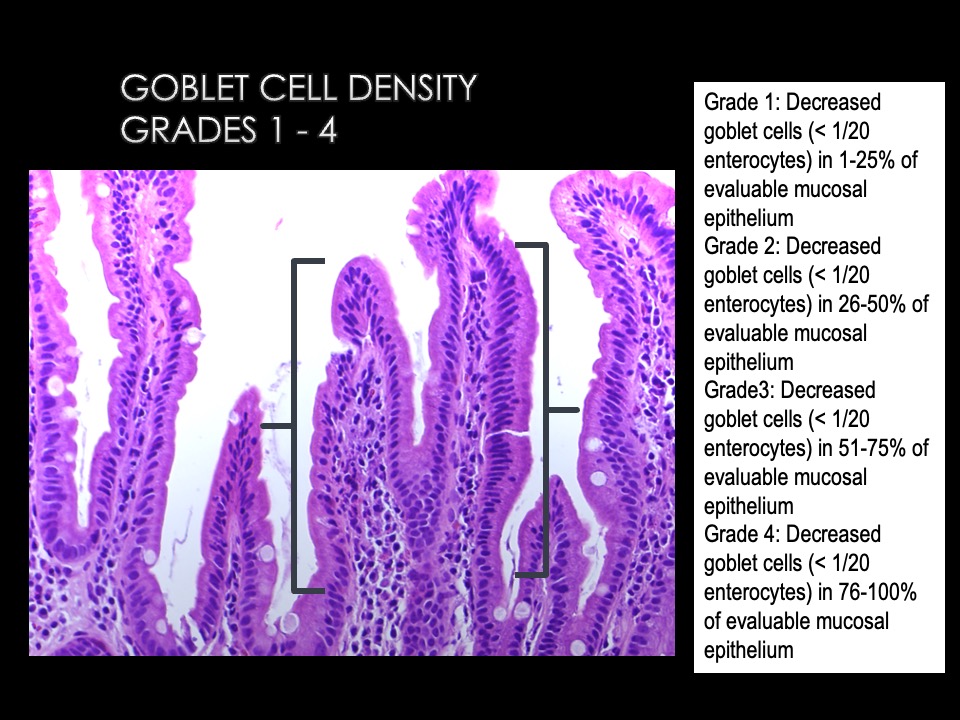

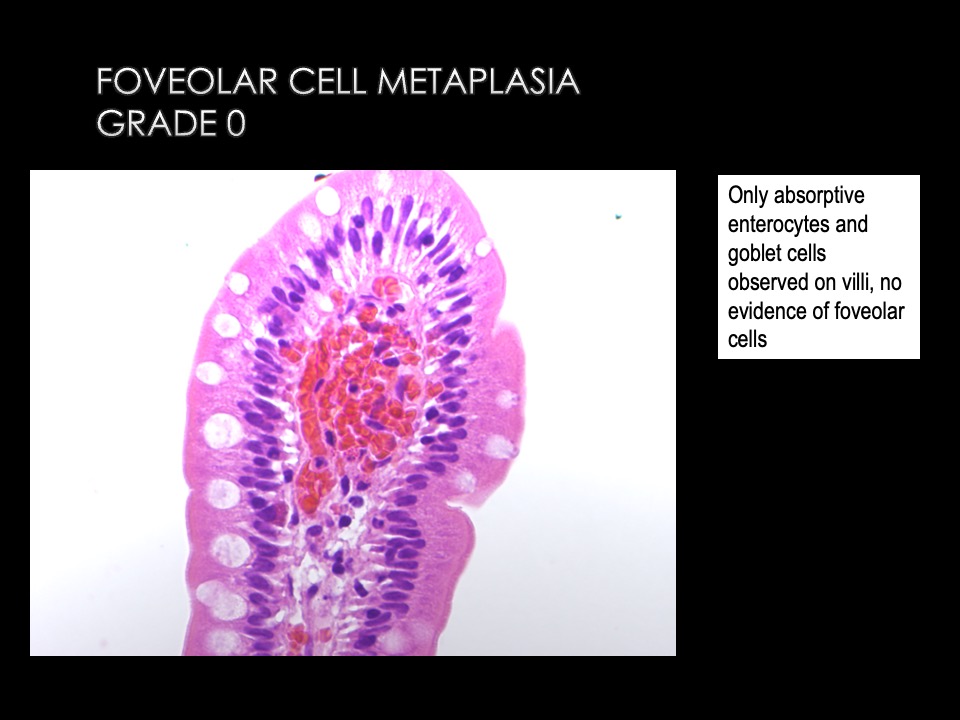

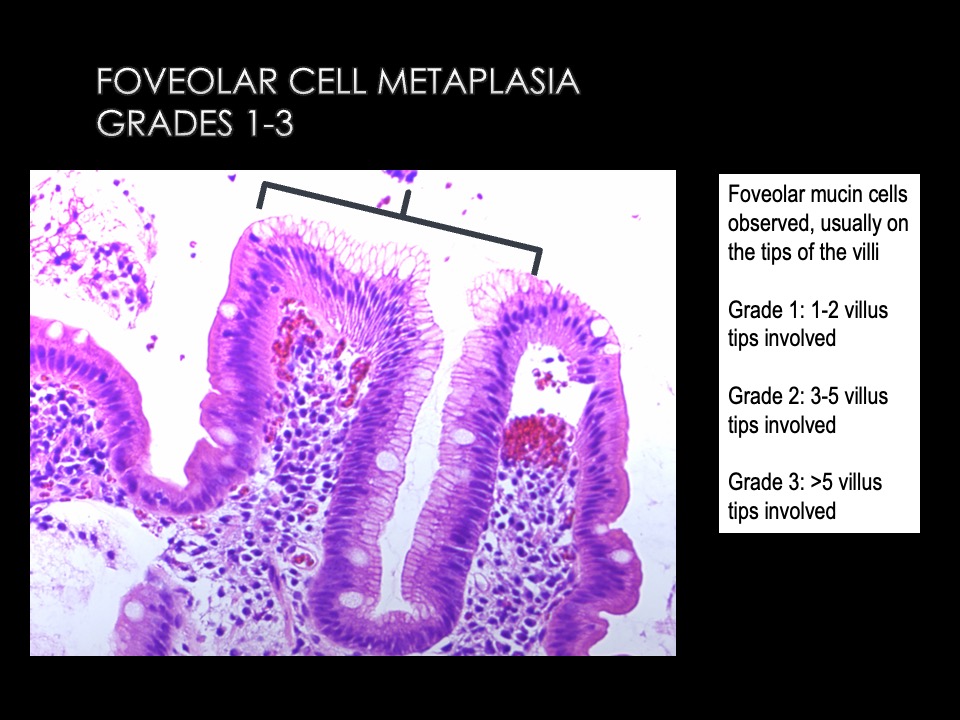

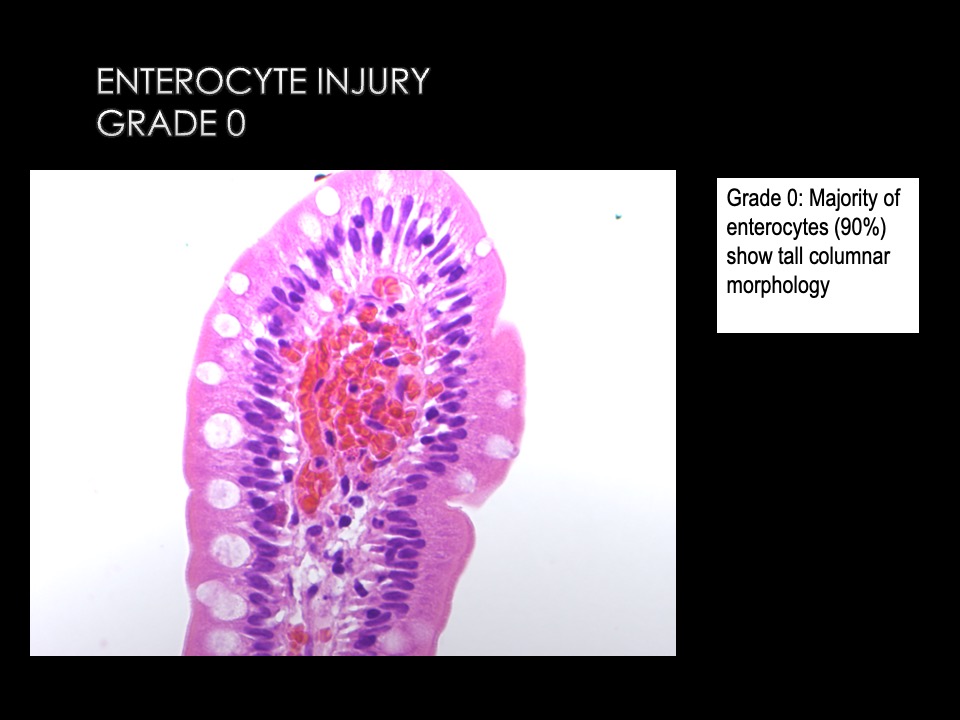

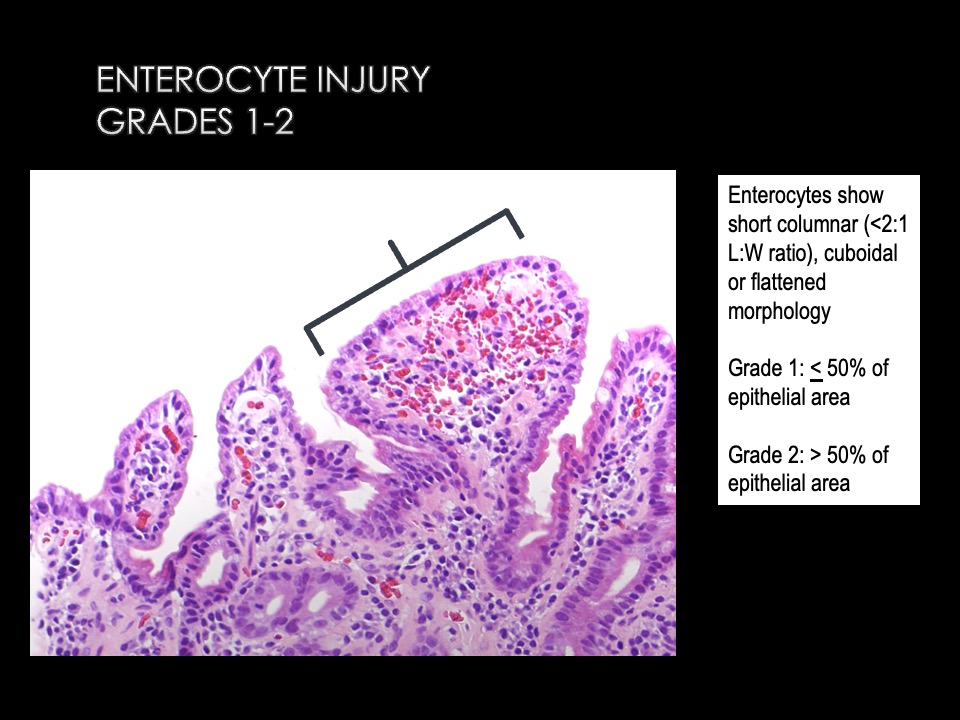

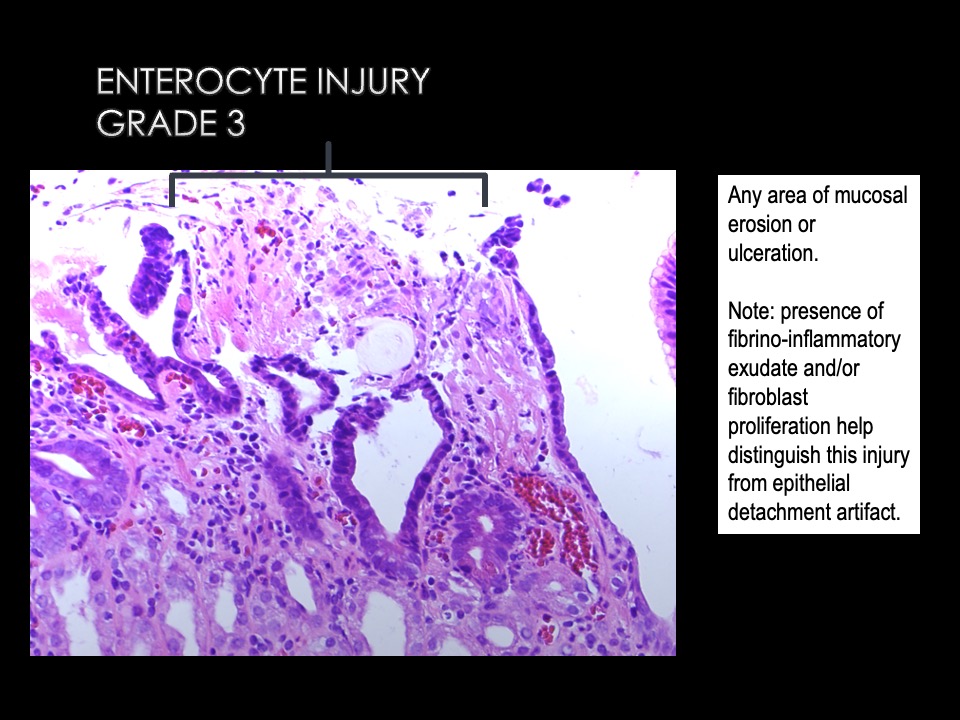

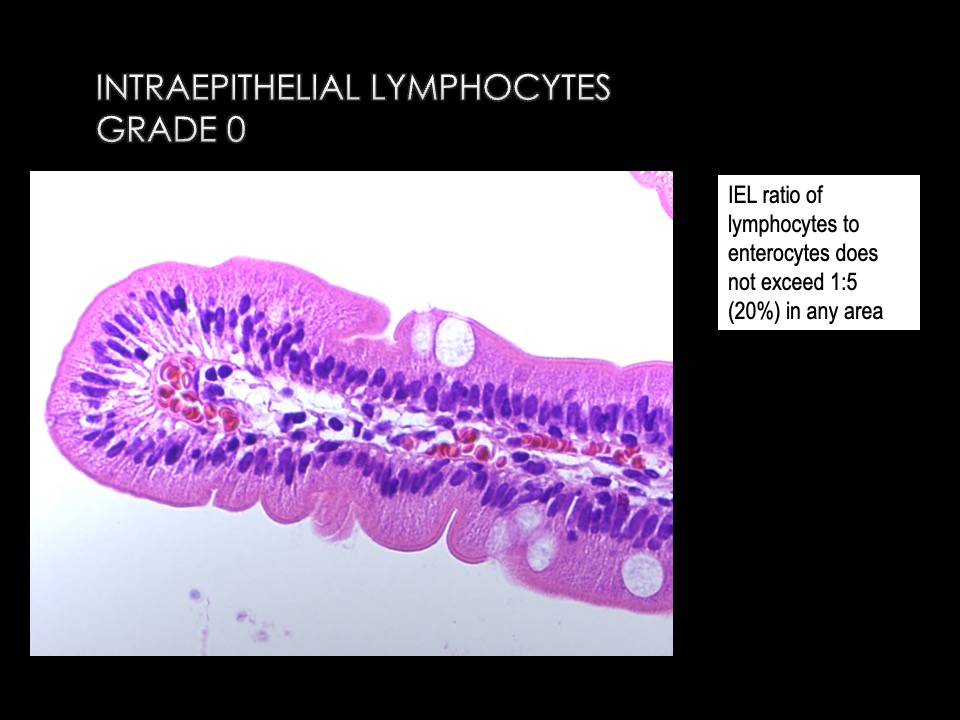

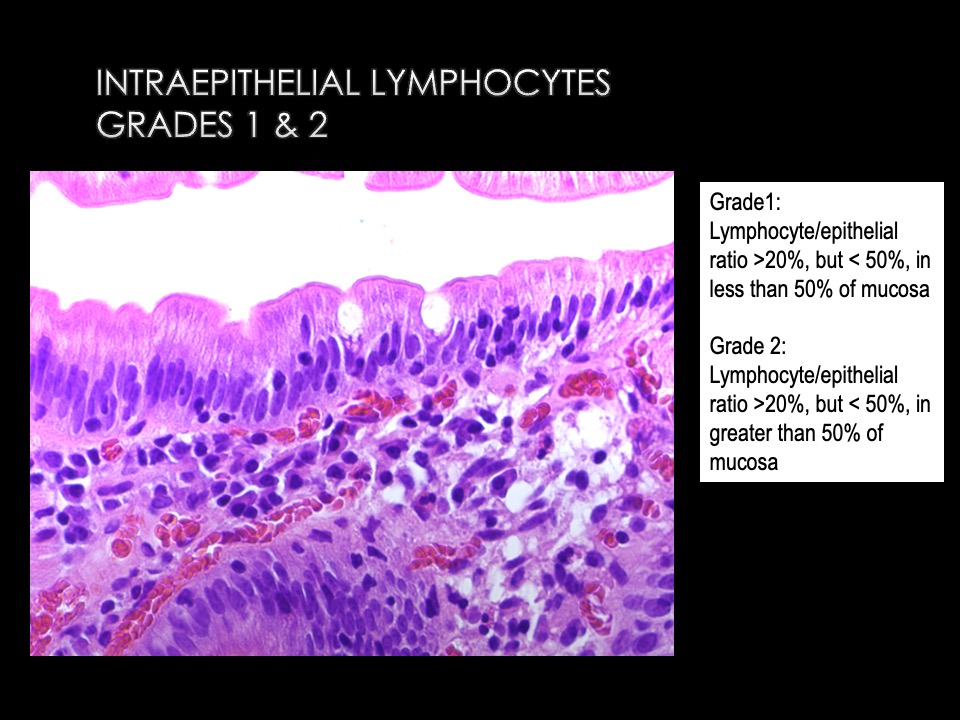

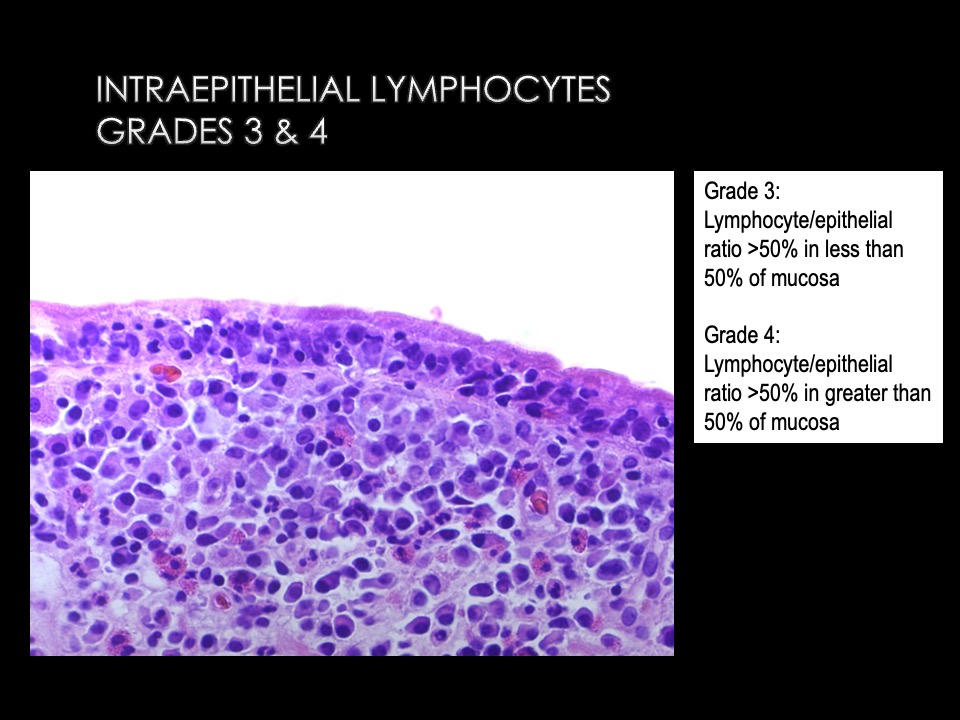

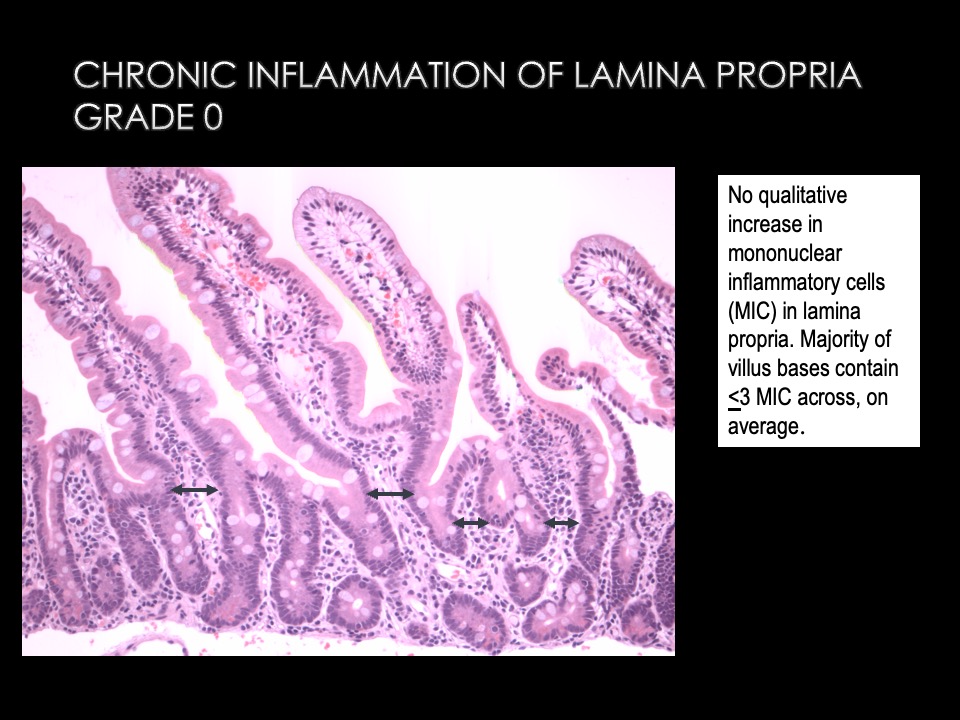

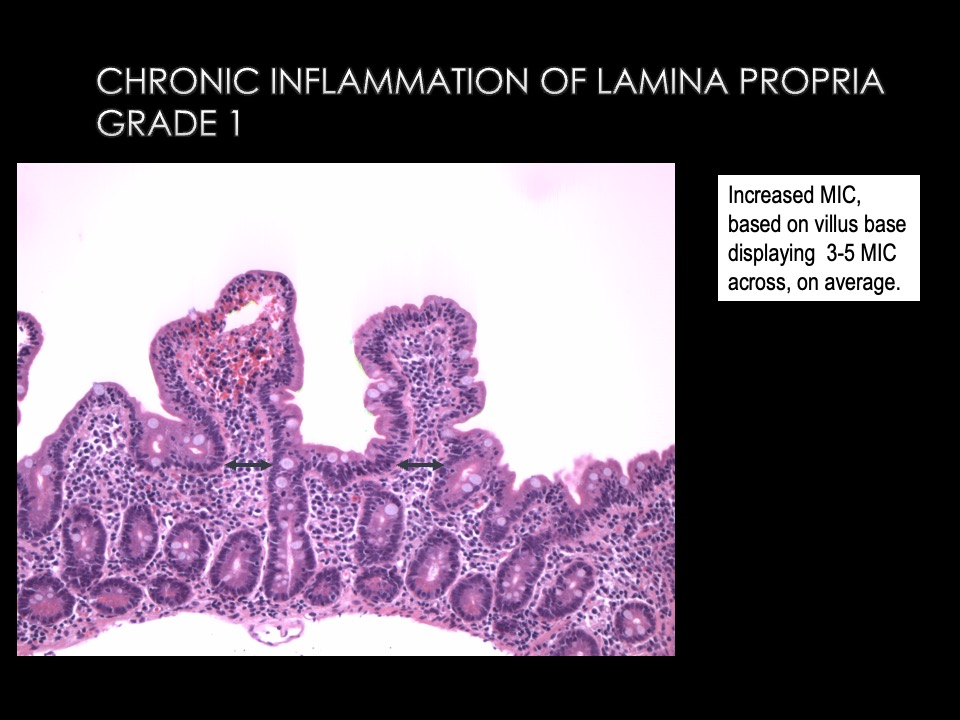

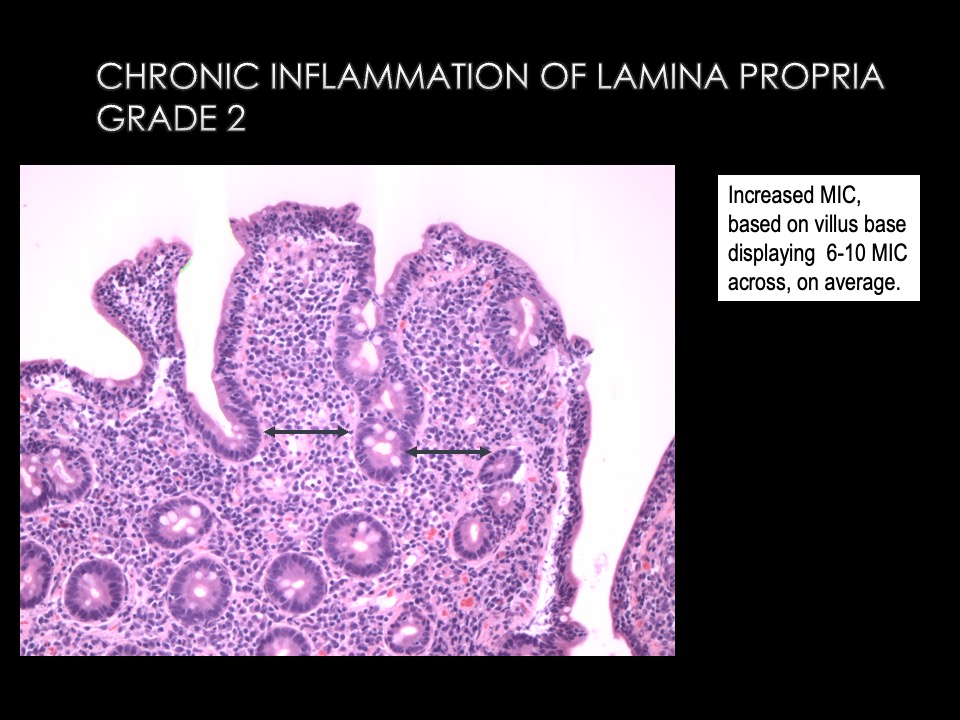

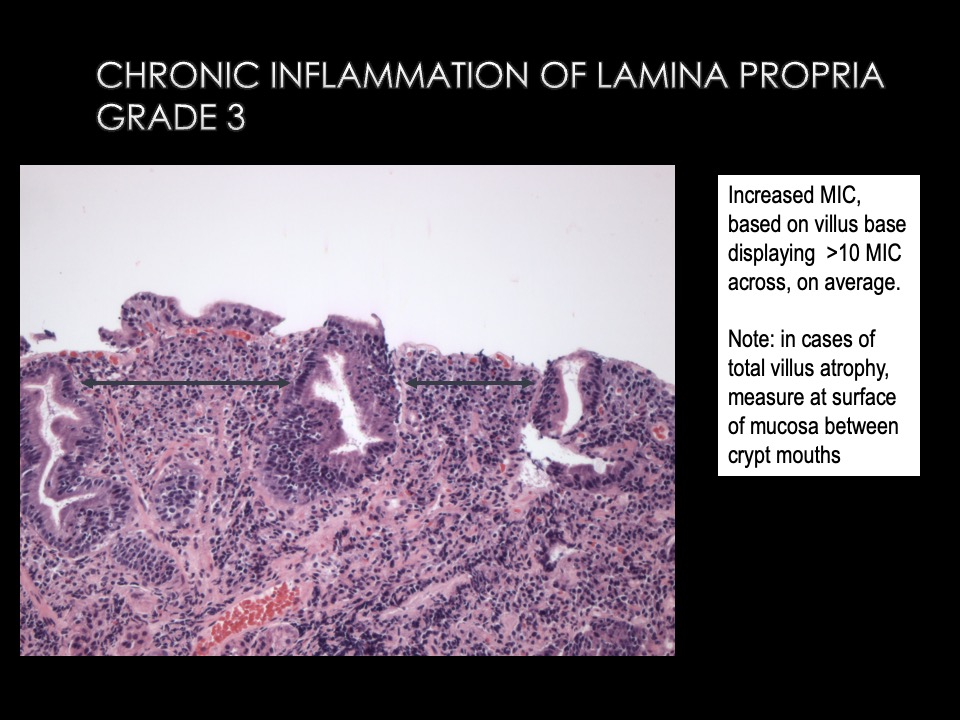

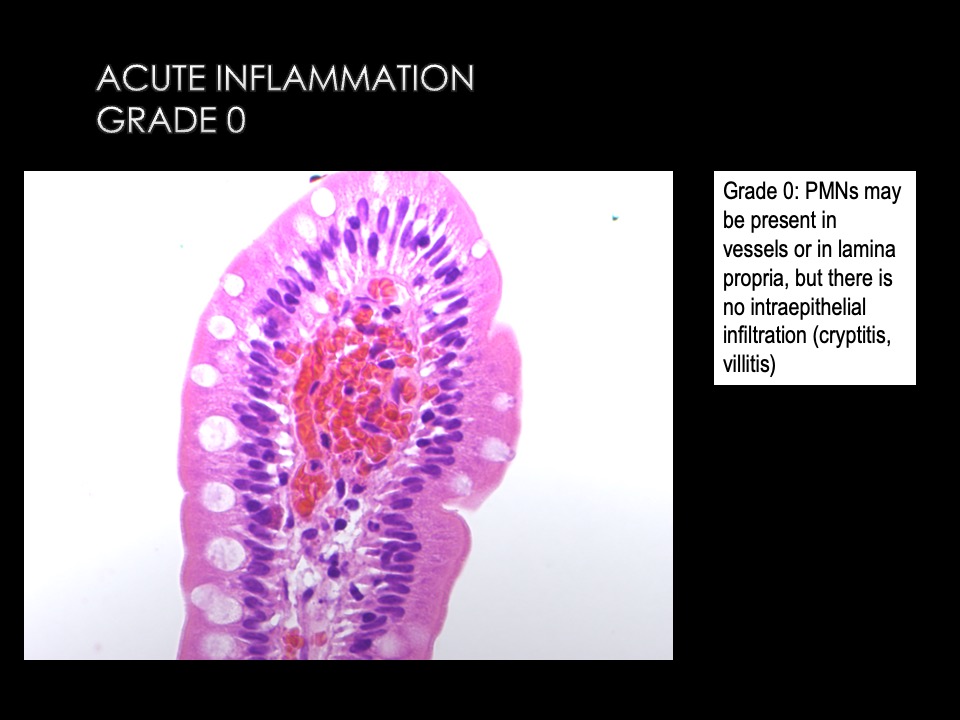

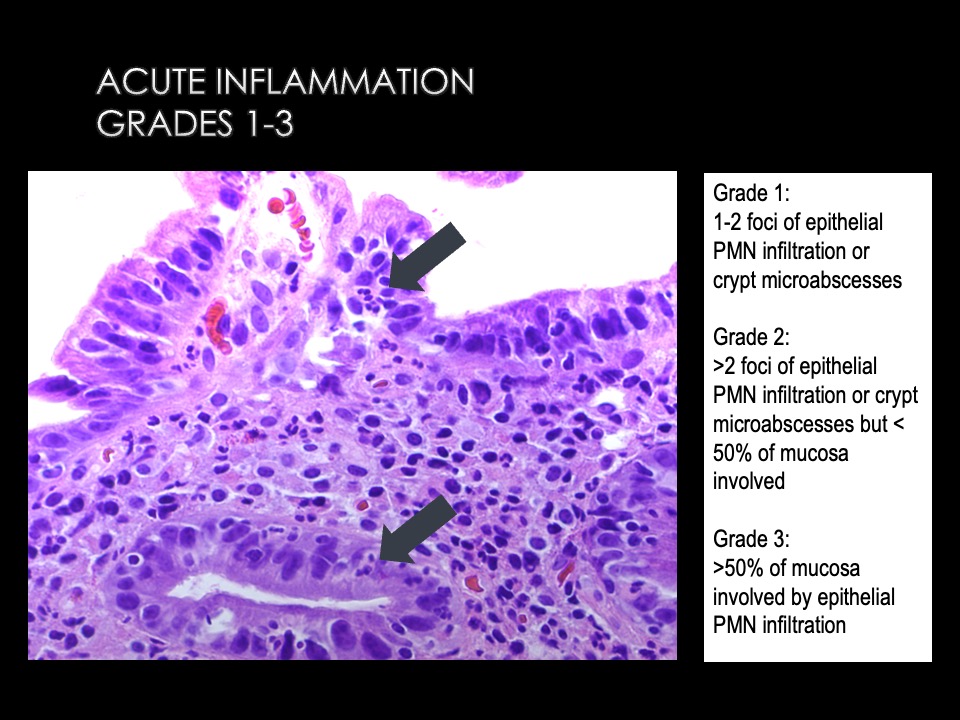

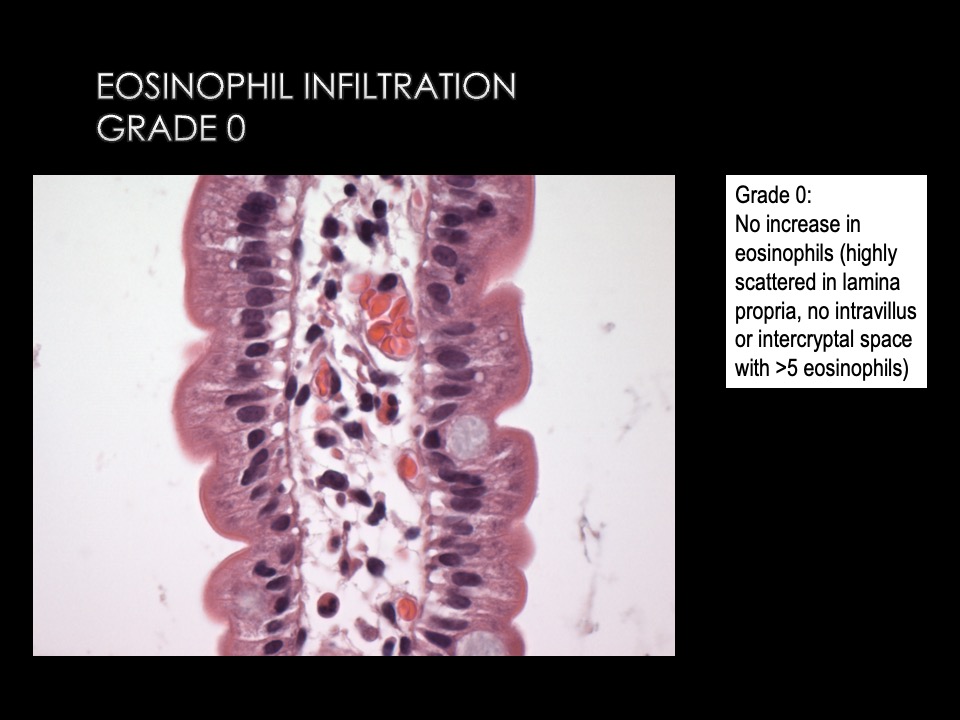

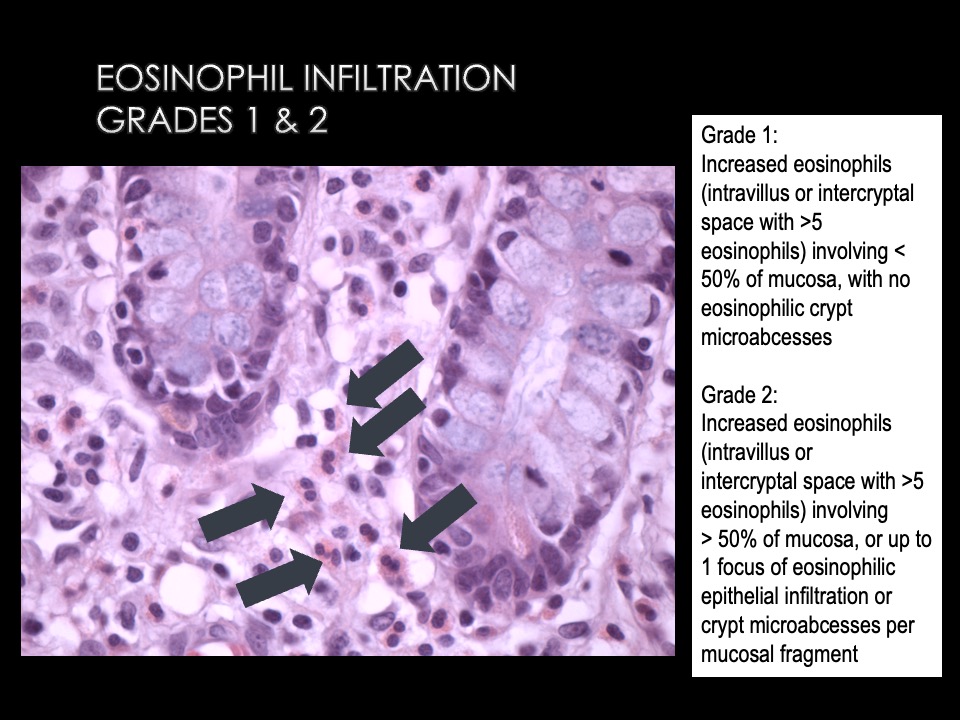

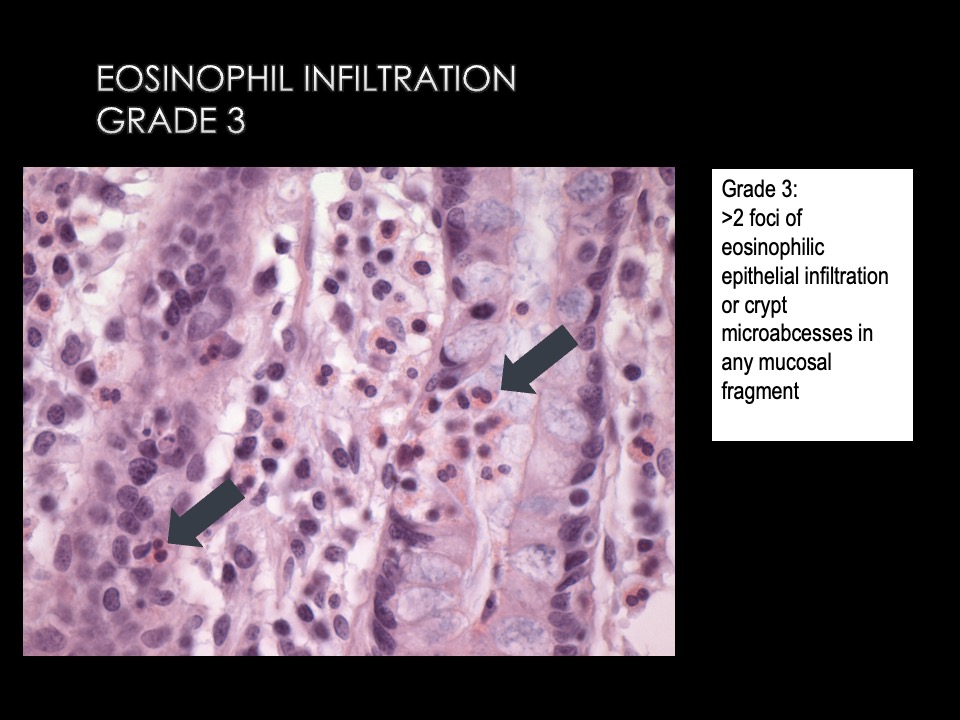

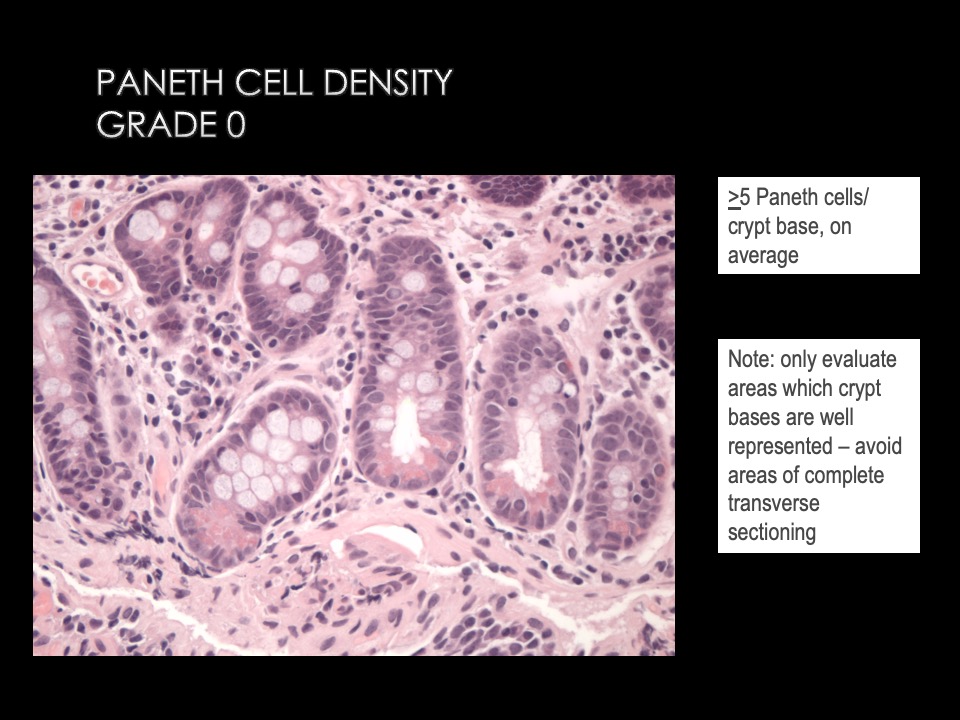

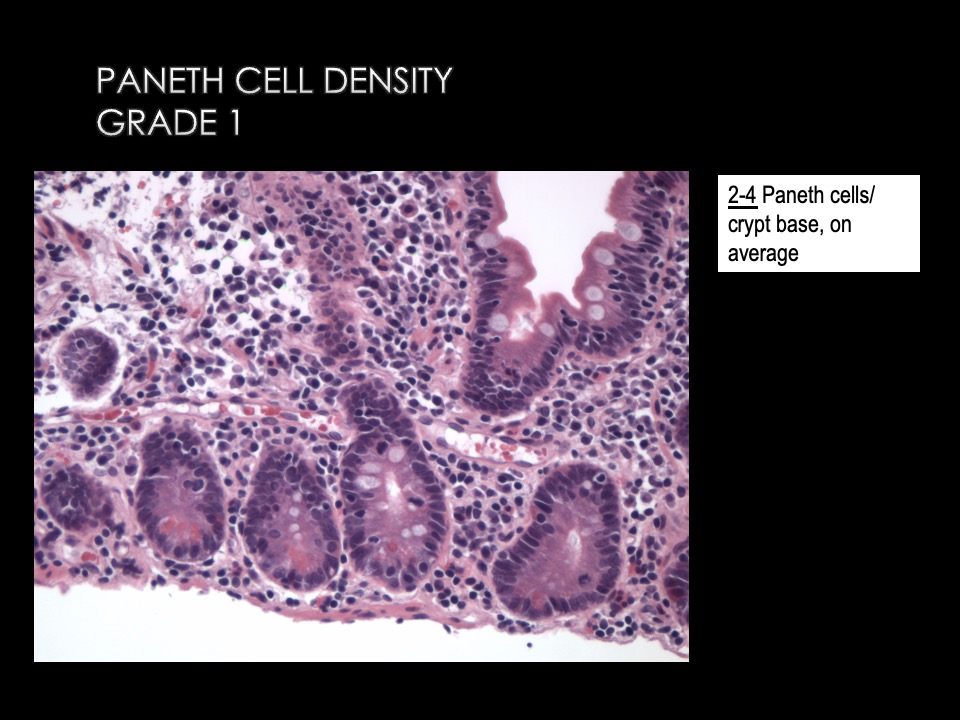

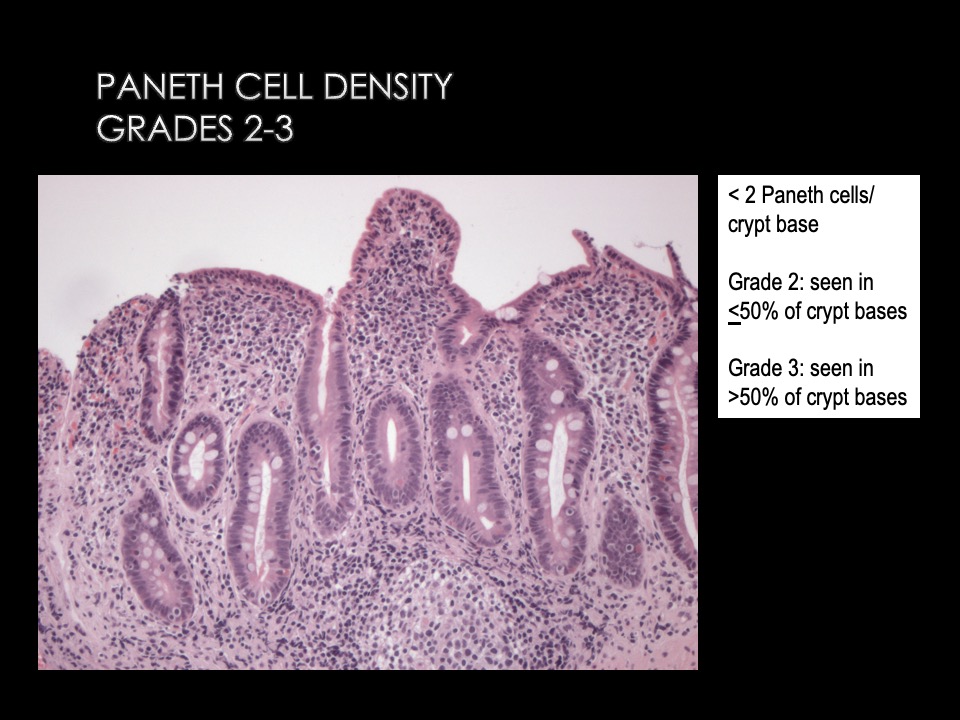

Supplement: Multimedia component 1 [file mmc1.zip › ajcnut_470_PAULKE~1_mmc1.DOC]
